# Targeted Codelivery of Prodigiosin and Simvastatin Using Smart BioMOF: Functionalization by Recombinant Anti-VEGFR1 scFv

**DOI:** 10.3389/fbioe.2022.866275

**Published:** 2022-03-24

**Authors:** Somayyeh Mirzaeinia, Sedighe Zeinali, Nediljko Budisa, Hamid Reza Karbalaei-Heidari

**Affiliations:** ^1^ Molecular Biotechnology Lab, Department of Biology, Faculty of Science, Shiraz University, Shiraz, Iran; ^2^ Department of Nanochemical Engineering, School of Advanced Technologies, Nanotechnology Research Institute, Shiraz University, Shiraz, Iran; ^3^ Department of Chemistry, Faculty of Science, University of Manitoba, Winnipeg, MB, Canada; ^4^ Institut für Chemie, Technische Universität Berlin, Berlin, Germany

**Keywords:** chemotherapy, BioMOF, prodigiosin, simvastatin, co-delivery

## Abstract

Biological metal-organic frameworks (BioMOFs) are hybrid compounds in which metal nodes are linked to biocompatible organic ligands and have potential for medical application. Herein, we developed a novel BioMOF modified with an anti-VEGFR1 scFv antibody (D16F7 scFv). Our BioMOF is co-loaded with a combination of an anticancer compound and a lipid-lowering drug to simultaneously suppress the proliferation, growth rate and metastases of cancer cells in cell culture model system. In particular, Prodigiosin (PG) and Simvastatin (SIM) were co-loaded into the newly synthesized Ca-Gly BioMOF nanoparticles coated with maltose and functionalized with a recombinant maltose binding protein-scFv fragment of anti-VEGFR1 (Ca-Gly-Maltose-D16F7). The nanoformulation, termed PG + SIM-NP-D16F7, has been shown to have strong active targeting behavior towards VEGFR1-overexpresing cancer cells. Moreover, the co-delivery of PG and SIM not only effectively inhibits the proliferation of cancer cells, but also prevents their invasion and metastasis. The PG + SIM-NP-D16F7 nanocarrier exhibited stronger cytotoxic and anti-metastatic effects compared to mono-treatment of free drugs and drug-loaded nanoparticles. Smart co-delivery of PG and SIM on BioMOF nanoparticles had synergistic effects on growth inhibition and prevented cancer cell metastasis. The present nanoplatform can be introduced as a promising tool for chemotherapy compared with mono-treatment and/or non-targeted formulations.

## Introduction

It was estimated that there were 19.3 million new cancer cases and 10 million cancer deaths worldwide in 2020 and this number is expected to reach 28.4 million by 2040, a 47% increase over the 2020 cases (Sung et al., 2021). The current treatment options for cancer are surgery, radiation, and chemotherapy which can be used alone or together (Huang et al., 2017). In chemotherapy, drugs travel through the body *via* bloodstream and destroy all cells, leading to an elevated toxicity in normal cells and the emergence of multiple drug resistance which can be described as a non-specific treatment approach.

Nanomedicine offers a platform of biocompatible and biodegradable systems that can be applied to enhance and control drug delivery (Zhao et al., 2013; Bregoli et al., 2016) to tumor sites and increase drug concentration in target tissues, leading to improvements in their solubility and release profile (Martinelli et al., 2019). A promising class of nanoparticles recently has attracted significant research interest is metal-organic frameworks (MOFs) which are composed of metal ions connected by organic linkers (Zhou H.-C. et al., 2012; Cook et al., 2013). They show a high porosity character which is ideal for increasing drug loading capacity in delivery systems (McKinlay et al., 2010).

In addition to MOFs, there are other porous nanoparticles such as silica-based NPs with unique pore structure, tunable surface and bulk chemistry, and carbon-based NPs including fullerenes, carbon nanotubes, graphene, and its derivatives with a very large length-to-diameter ratio (Singh et al., 2017; Patel et al., 2019). Compared to silica-based NPs, MOFs are more flexible, provide better biodegradability, and offer a wider range of pore sizes. Carbon nanotubes have a confined ability of modification and less porous structures than MOFs (Saeb et al., 2021). MOFs can be synthesized in a wider range of diverse morphologies including spherical, cubic, hexagonal, ellipsoidal, and octahedral, which facilitates the acceptance of various molecules (Maity and Polshettiwar, 2018). They are not only promising due to their physicochemical properties but also due to their ability to provide a wide range of interactions, such as physical interaction and π–π interactions on the surface or inside the porosities with guests (cargos) molecules (Saeb et al., 2021).

BioMOFs are a new subclass of MOFs that use biological molecules including nucleobases, amino acids, peptides, proteins, porphyrins and, saccharides as linkers which create a biocompatible nanocarrier (Wang et al., 2020). The carboxyl-O atom and/or amino-N atom of amino acids are ideal ligands for coordination with metal ions and the preparation of bio-MOFs (Cai et al., 2019).

Among various targeting ligands (Sun et al., 2014; Pérez-Herrero and Fernández-Medarde, 2015), monoclonal antibodies (mAb) are widely used to actively target tumor cells due to their high specificity. To overcome the limitations of full-size mAb such as their size, complexity, post-translational modifications, and poor penetration into cancer cells, antibody fragments including Fab, scFv and VHH have been introduced in recent years (Weisser and Hall, 2009). ScFv as one of the most popular antibody fragments has improved therapeutic potential due to its smaller size, low immunogenicity and low production cost (Ahmad et al., 2012). The scFvs contain the antigen binding site comprising the variable heavy (VH) and variable light (VL) domains of a full-length mAb linked by a flexible polypeptide linker (G4S)_3_. Due to an intra-domain disulfide bond (Wörn and Plückthun, 2001), scFv expression usually requires an oxidizing environment, as found in the eukaryotic endoplasmic reticulum (Miller et al., 2005) or bacterial periplasm (Skerra et al., 1991).

To achieve the cost-effective expression of scFv, prokaryotic hosts like *Escherichia coli* (*E. coli*) are preferred. However, the reducing environment of bacterial cytoplasm leads to the formation of inclusion body of expressed scFv (Sarker et al., 2019). So far, the use of partner proteins or peptides as fusion components of scFv has been reported. It has also been reported that cytoplasmic expression of scFv as a fusion with maltose-binding protein (MBP) results in a soluble and functional MBP-ScFv fusion protein (Bach et al., 2001). Also, the use of the CyDisCo system allows soluble expression of disulfide-bonded proteins in the cytoplasm of *E. coli*. The CyDisCo system relies on the co-expression of a protein with a sulfhydryl oxidase enzyme, Erv1p, and a protein disulfide isomerase (PDI) chaperone to enhance proper folding (Gaciarz et al., 2016).

Vascular endothelial growth factor receptor-1 (VEGFR-1) is an ideal candidate for targeting cancer cells because it is frequently overexpressed in various human cancers such as brain, breast, prostate, kidney, ovarian, lung and bladder (Goel and Mercurio, 2013). VEGFR-1 (fms-like tyrosine kinase-1, Flt-1) is a tyrosine kinase receptor (TKR) that binds to VEGF-A, VEGFB, and placental growth factor (PlGF) ligands (Roskoski, 2008; Takahashi and Bulletin, 2011) and induces receptor dimerization, tyrosine autophosphorylation, transphosphorylation, and signaling proteins docking (Roskoski, 2008; Cao, 2009). Therefore, by producing a recombinant scFv against the VEGFR1 receptor, cancer cells can be targeted.

Prodigiosin (PG) is a red pigment from *Serratia marcescens* (Rastegari and Karbalaei-Heidari, 2016), that exhibits anticancer activity in eukaryotic cells due to its proapoptotic action, cleavage of double stranded DNA and disruption of the pH gradient (Pérez-Tomás and Vinas, 2010; Anwar et al., 2020). On the other hand, cholesterol is an essential component of the cellular membrane, it accumulates in cancer cells and tumor tissues and is involved in various cellular processes such as cell growth, proliferation, and migration. In cancer patients undergoing chemotherapy, blood cholesterol levels increase leading to increased cell resistance to chemotherapy drugs (Hilvo et al., 2011; Cruz et al., 2013). According to the results of several studies, statins can reduce risk of tumor aggressiveness and mortality in cancer cells (Allott et al., 2016). Simvastatin (SIM) has been shown to have antiproliferative and apoptotic effects on numerous cancers by arresting cell cycle, inhibiting tumor metastasis and inducing apoptosis (Koyuturk et al., 2007; Sun et al., 2015; Balata et al., 2016). Thus, the combination of Simvastatin and Prodigiosin may act synergistically to inhibit tumor progression. Since each agent acts on different pathways of cell metabolism, simultaneous administration of two or more therapeutic agents enhances their ability to stop tumor cell proliferation and reduce the most prevalent behavior of cancer cells i.e., drug resistance (Wang and Huang, 2020).

Therefore, the aim of this study was to design a new smart co-delivery platform that can efficiently load drugs and target the specific tumor area. To this end, we prepared Ca-Gly BioMOF nanoparticles modified with an anti-VEGFR1 scFv fragment and determined the toxicity, apoptotic effects and cellular uptake by cellular assays and fluorescence microscopy on cancer cell lines. For this purpose, the synthesized Ca-Gly BioMOF was coated with maltose-NH_2_. The anti-VEGFR1 scFv fragment of the VEGFR-1 antibody (D16F7) was recombinantly produced as a fusion protein with MBP in the presence of a co-expression system in *E. coli* Bl21 (DE3). PG and SIM were loaded into the BioMOF surface modified with the recombinant scFv of D16F7 antibody to establish a smart targeting system. We demonstrated that the PG can enhance therapeutic potential by suppressing proliferation, inhibiting cancer cells migration and inducing apoptosis when applied in combination with SIM in a synergistical manner.

## Experimental Details

### Materials, Strains, and Cell Lines

Chemicals were provided by either Merck or Sigma-Aldrich. Restriction enzymes and T4 ligase were purchased from Fermentas (Vilnius, Lithuania). The helper plasmid PMJS205 was kindly provided by Prof. Lloyd Ruddock. *Escherichia coli* BL21 (DE3) was from Invitrogen (Thermo Fisher Scientific) and the pMAL-c2X plasmid was provided from Addgene (#75286). The optimized DNA sequence encoding the D16F in pUC57 was synthesized by Genscript company. Dulbecco′s modified Eagle′s medium (DMEM), trypsin-EDTA, Penicillin/Streptomycin and fetal heat-inactivated bovine serum (FBS) were purchased from Gibco^®^ (Gaithersburg, USA). Cancerous cell lines including MCF-7 (human breast cancer), LnCap (human prostate cancer), U87MG (human glioblastoma) and human skin fibroblast (HSF) were obtained from Department of Cell Bank, Pasteur Institute of Iran.

### BioMOF Synthesis and Maltose Coating

#### Synthesis of BioMOF (Ca-Gly)

Four mmol *Glycine* in 5 ml methanol was mixed in a beaker to disperse the linker. Then, 2 mmol CaCl_2_ and 2 mmol NaOH in 5 ml water were added to the suspension. The suspension was mechanically stirred for 1 hour to give a clear colorless solution and then transferred to a round bottom flask. The reaction was refluxed at 100°C for 15 h. After cooling to room temperature (RT), the white precipitate was collected by centrifugation and washed several times with water/EtOH. The Ca-Gly crystals were dried in an oven at 60°C (Kathalikkattil et al., 2015).

### Preparation of Ca-Gly-COOH NPs

2.5 g succinic anhydride was added to the solution of 500 mg Ca-Gly NPs in 120 ml tetrahydrofuran. The dispersion solution was refluxed for 12 h. The obtained Ca-Gly-COOH NPs were washed several times with deionized water and ethanol and dried in an oven (Bi et al., 2018).

### Synthesis of Maltose–NH_2_

0.004 mol of maltose and 0.004 mol of NH_4_HCO_3_ were added to 20 ml of an aqueous NH3 solution. This solution was heated at 42°C for 36 h, concentrated to half the volume, and then lyophilized (Zhou L. et al., 2012).

### Synthesis of Ca-Gly-Maltose NPs

500 mg of maltose–NH2 was added to 50 ml of deionized water containing 100 mg Ca-Gly-COOH NPs. 0.5% (w/v) cyanamide and 0.5% (w/v) 1,6-diaminohexan were added to the solution and incubated overnight at RT. After the reaction, the Ca-Gly-Maltose NP was washed several times with deionized water and ethanol. The product was dried in an oven.

### BioMOF Characterization

The crystal structure of the synthesized NPs was analyzed by Powder X-ray diffraction (PXRD) analysis (Bruker, D8 advance, Germany). Morphology and size of nanoparticles were studied by SEM (TESCAN Vega three microscope) at an accelerating voltage of 20 kV and samples were sputter*-*coated with gold. The carboxylation and maltose surface modification of Ca-Gly-Maltose NPs (BioMOF) were analyzed by Fourier transform infrared (FT-IR) spectroscopy (Perkin Elmer RXI*,* United States). The thermal behavior was investigated by a Thermogravimetric analyzer (TGA). Surface area measurements were analyzed using the BET instrument (BELSORP Mini II). The hydrodynamic diameter (Dh) and ζ-potential measurement were performed with a DLS instrument (HORIBA SZ-100).

### Drug-Loading

Prodigiosin (PG) and Simvastatin (SIM) loading was performed by adsorption method for both, alone and in combination. Briefly, 1 mg of Ca-Gly-Maltose NPs were dispersed in 10 ml of PBS buffer solution with a pH∼7.4 for 10 min. Drug loading was performed by iteratively adding drug to the nanoparticle solution at 10 min time intervals while stirring on a rotator (30 rpm) at 25°C.

To estimate the encapsulation efficiency (EE), the protocol previously reported was followed (Rastegari et al., 2017). Briefly, a certain amount of drug-loaded NPs was collected using a centrifuge and the aqueous phase was discarded. After adding 10 ml of methanol, the mixture was shaken for 10 min to completely re-dissolve the entrapped drug. The supernatant was assessed by a spectrophotometer at 535 nm for PG and at 239 nm for SIM. (Shah and Pathak, 2010; Rastegari and Karbalaei-Heidari, 2016). The drug encapsulation efficiency (EE%) and loading capacity (LC) were calculated by following equations:

$$EE\left(\%\right)=\frac{\mathrm{drug initial weight (mg) - unloaded drug in aqueous phase(mg)}}{\mathrm{drug initial weight(mg)}}\times 100$$

$$LC=\frac{\mathrm{Drug initial weight (mg) - unloaded drug in aqueous phase(mg)}}{\mathrm{Nanoparticle weight}\left(\mathrm{mg}\right)}$$

### *In-vitro* Drug Release

*In vitro* drug release from the Ca-Gly-Maltose nanoparticles were monitored by UV-visible spectrophotometer at 239 nm and 535 nm for PG and SIM, respectively. In order to simulate the biological condition of lysosome, tumor microenvironment and physiological pH of blood stream, the drug release profile of PG and SIM from the newly designed BioMOF was investigated in PBS solution at 37°C during 2 h at pH 5.0, 6.5 and 7.4.

1 mg of drug loaded nanoparticles were dissolved in 10 ml phosphate buffered saline and dispersed with sonication for 5 min. At specified time points, the nanoparticles were collected by centrifuge and 500 µl of the aqueous solution was mixed with acidified ethanol in 1:1 ratio. Concentration of PG and SIM in supernatant was measured by spectrophotometer in a method as described above.

### Gene Synthesis and Cloning

A gene encoding for D16F7 scFv was chemically synthesized after codon optimization to be expressed in bacterial host and ordered as an insert in the pUC57 plasmid. The gene consisted of the sequences encoding the anti-VEGFR-1 antibody variable heavy (VH) and variable light (VL) chains (Sanjuan et al., 2016), a peptide linker (Gly4Ser)3 between them, and a hexa-histidine tag at the C terminus (VL-(Gly4Ser)3-VH-H6) (Supplementary Figure S1). The synthetic gene was then cloned into the expression plasmid, pMAL-c2X which contains a maltose-binding protein (MBP) partner upstream of multiple cloning sites (Alexandrov et al., 2001). The correctness of the new construct, pMAL-c2X-D16F7, was confirmed by DNA sequencing.

### Expression and Purification of scFv D16F7

The helper plasmid pMJS205, and the recombinant construct (pMAL-c2X-D16F7) were co-transformed into *E. coli* BL21 (DE3) and grown on LB-agar plate containing 100 μg/ml ampicillin and 35 μg/ml chloramphenicol. A single colony was transferred to LB medium containing both antibiotics overnight at 37°C. The preculture was used to inoculate ZYM-5052 autoinduction medium (Studier, 2005) with the appropriate antibiotics and incubated at 25°C and 250 rpm overnight. Cultures were harvested by centrifugation and pellets were resuspended in a lysis buffer containing 50 mM sodium phosphate buffer, pH = 7.4, 150 mM NaCl and 1% triton x-100. To purify the overexpressed MBP-scFv fusion protein, the soluble fraction of cell extract was loaded onto a Ni-NTA agarose column (1 ml). The partially purified MBP-scFv protein was dialyzed against 20 mM Tris -HCl (pH 7.5), and the homogeneity of the sample was analyzed by sodium dodecyl sulfate-polyacrylamide gel electrophoresis (SDS-PAGE) in 12.5% acrylamide gels.

### Binding of MBP-D16F7 scFv With Ca-Gly-Maltose NPs

Immobilization of MBP-D16F7 scFv on maltose-coated BioMOF was performed using 1:6, 1:8, 1:10, and 1:20 ratios of MBP-scFv and Ca-Gly-Maltose NPs. The MBP-scFv fusion protein was incubated with 2 mg Ca-Gly-Maltose NPs overnight at 4°C. Unbound MBP-scFv was monitored by centrifugation of the nanoparticle solution and analysis of the supernatant by Vis-UV spectrophotometer at 280 nm (*ε* = 113,,220 M^−1^ cm^−1^) (Liu et al., 2009).

### Cytotoxicity Assays

All cell lines were cultured in modified Dulbecco’s Eagle Medium (DMEM) supplemented with 10% fetal bovine serum (FBS), 1% penicillin/streptomycin and maintained at 37°C and 5% CO_2_ in a humidified atmosphere in cell incubator. The MTT assay was used to assess the cytotoxicity of free drugs and various nanoformulations against MCF-7, LnCap, U87MG and human skin fibroblast (HSF) cell lines. Cells (1 × 10^4^ cells/well for MCF-7 and U87MG, 1.5 × 10^4^ cells/well for LnCap and 1.2 × 10^4^ cells/well for fibroblast) were seeded in 96-well plates and incubated overnight. After 24- and 48-h incubation, the cells were washed twice with PBS and MTT was added at final concentration of 0.5 mg/ml. After 4 h of incubation at 37°C, the culture media were removed, and the formazan crystals were solubilized in a solution containing 40.0% (v/v) DMF, 16.0% (w/v) SDS and 2% (vol/vol) glacial acetic acid pH∼4.7. Then, the absorbance was measured using a SPECTROstar Nano (BMG Labtech, Germany) microplate reader at 570 nm after background correction at 630 nm. The percentage of cell viability was calculated as follow:

$$viability\left(\%\right)=\frac{A_{T}\;\left(sample\right)}{A_{T}\;\left(control\right)}\times 100$$

$${\text{A}}_{\text{T}}={\text{A}}_{570}-{\text{A}}_{630}$$

### *In vitro* Cellular Uptake

Cellular uptake of the formulations in cells was determined by fluorescence microscopy. Briefly, 2.5 × 10^4^ cells of MCF-7, LnCap, U87MG and HSF were seeded in 48 well plates and incubated at 37°C for 24 h. The culture media were then replaced with fresh medium containing IC_20_ concentrations of free PG-SIM, PG + SIM-NP and PG + SIM-NP-D16F7 for periods of 30, 90 min, and 24 h. After incubation, cells were washed three times with PBS and fixed with 4% formaldehyde for 20 min. Then, the cells were stained with 300 nM DAPI for 5 min and washed three times. Finally, cells were washed three times with PBS and observed with a Florescence microscope (Olympus; IX51).

### *In Vitro* Scratch Assay

MCF-7 cells were seeded at a concentration of 4 × 10^4^ cells/ml in 24 well plates and allowed to grow to a confluence level of 70–80%. Then, wounds were created in each well using a 10 µl pipette tip. The debris was removed by washing with PBS. IC_20_ concentrations of the above formulations were added and incubated for 48 h. Images were taken immediately after the incision as well as 24 and 48 h after incubation. Migration distance was measured using ImageJ software and wound healing rate was calculated with the following equation:

$$\mathrm{Scratch}\;\mathrm{healing}\;\mathrm{rate}=\left(W_{0}-W\right)/W_{0}\times 100$$

where W_0_ is wound width of sample at 0 h and W are the wound width after 24or 48 h (Li et al., 2019).

### Statistical Analyses

Data in this study were analyzed using the software GraphPad Prism, version 8.3.0 (GraphPad, San Diego, CA, United States). Comparison between groups was made using the one way with Dunnett’s or two-way ANOVA with Tukey test. Differences with *p*-values less than 0.05 indicated significance. Combination index (CI) values were determined by CompuSyn software version 1.0 (freeware, The CompuSyn, Inc, Paramus, NJ, United States).

## Results

### Preparation and Physicochemical Characterization of BioMOF

**FIGURE 1:**
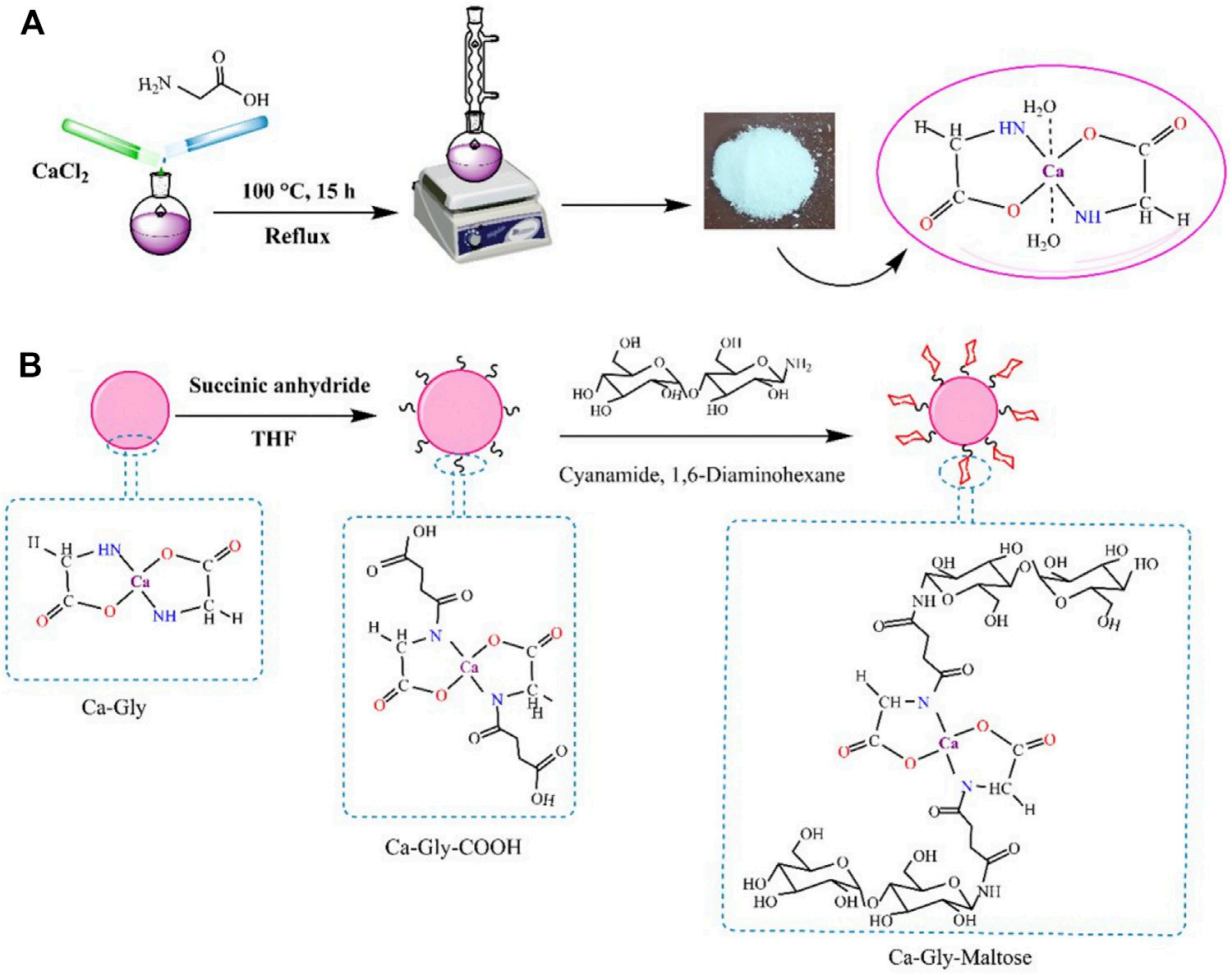
Schematic illustration of the **(A)** Synthesis pathway of Ca-Gly and **(B)** surface modification and fabrication of Ca-Gly-Maltose.

In this study, we synthesized a non-toxic Ca-based BioMOF, which is named Ca-Gly. As shown in Figure 1, Ca^2+^coordinates with the carboxyl (-COOH) and amino (-NH_2_) groups of glycine (Yin et al., 2017). Water molecules are also present in the crystal lattice and the Ca^2+^ ions are associated with the oxygen of water. The BioMOF surface was modified by treatment with succinic anhydride containing carboxylic groups. Then, reacted with the amine group of the maltose–NH_2_ to obtain Ca-Gly-Maltose nanoparticles (Figure 1).

**FIGURE 2:**
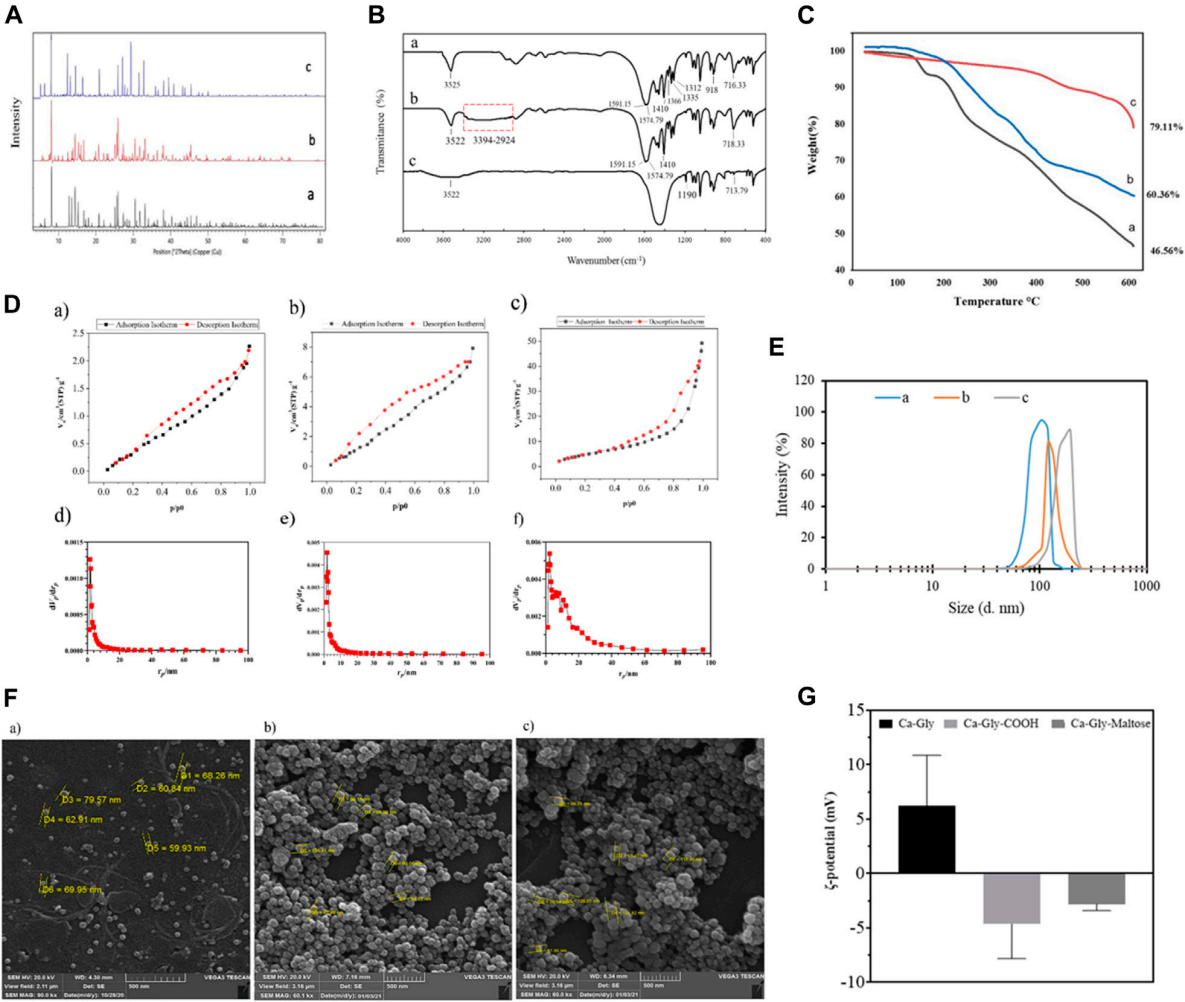
**(A)** XRD Pattern, **(B)** FTIR spectra, **(C)** TGA analysis, **(D)** N2 adsorption isotherm at 77 K of a) Ca-Gly, b) Ca-Gly-COOH, c) and Ca-Gly-Maltose after outgassing at 150°C; and BJH pore volume distribution curves of d) Ca-Gly, e) Ca-Gly-COOH, f) and Ca-Gly-Maltose**, (E)** Hydrodynamic size distributions, **(F)** Zeta potential and **(G)** SEM images of a) Ca-Gly, b) Ca-Gly-COOH, c) and Ca-Gly-Maltose.

To investigate the crystalline structure and ensure the synthesis of Ca-Gly, Ca-Gly-COOH, Ca-Gly-Mal, XRD analysis was performed (Figure 2A). The strong peaks at small angles (2ɵ) prove that ample pores are present in the synthesized BioMOF structure. As observed, the main peaks are at 2ɵ values equal to 5.63, 6.49, 8.1, 14.4, 15.23, 16.7, 18.05, 20.79, 22.08, 25.65, 30.47, and 31.08° indicating the crystalline nature of the nanoparticles. The XRD patterns confirmed that the crystalline structure of BioMOF NPs was not changed after surface modification. In Ca-Gly-Maltose, the 29° peak associated with maltose was enhanced.

The average crystallite sizes were determined to be 287, 325, and 319°Afor Ca-Gly, Ca-Gly-COOH, Ca-Gly-Mal using the Debbie Scherrer equation with X’pert software.

The FT-IR spectra of the synthesized Ca-Gly, Ca-Gly-COOH and Ca-Gly-Mal are shown in Figure 2B. The observed peak at 716 cm^−1^ is associated with Ca-O vibration, which is also seen with a shift in Ca-Gly-COOH and Ca-Gly-Maltose spectra. The peaks at 1,312, 1,335 and 1,366 cm^−1^ belong to the C-N bond in the amino acid which has been disappeared in the Ca-Gly-Maltose nanocarrier. Compared with the spectrum of Ca-Gly, the new broad peak in the wavenumber region of 2,964–3,448 cm^−1^ (the carboxyl group) and an enhanced peak at 1,557 and 1,591 cm^−1^ (the amide group) and a peak at 1,410 cm^−1^ (for OH of the carboxylic acid group) in the spectrum of Ca-Gly-COOH revealed the successful modification of carboxyl groups on the surface of Ca-Gly (Bi et al., 2018).

The thermal stability of the prepared formulations was evaluated by thermo gravimetric analysis (TGA). As shown in Figure 2C, weight loss occurred in three stages. In the first stage, the lattice water molecules trapped in the pores were lost, with 7% of the weight Ca-Gly evaporating in the temperature ranges of 129–163°C. In the temperature ranges of 163–245 °C, coordinated water molecules had disappeared. The evaporation of the water molecules in this stage resulted in a weight reduction of 20% of Ca-Gly. Finally, in the third stage, from 245°C glycine degradation occurred (Kathalikkattil et al., 2015) and up to 600°C, Ca-Gly lost about 53.44% of its weight. The stability of Ca-Gly-COOH, and Ca-Gly-Maltose increased, so the Ca-Gly-COOH lost 30% of the weight at 150–427°C and 39.64% of the total weight up to 600°C. For Ca-Gly-Maltose, the weight loss up to 600°C was about 21%.

The BET surface areas of Ca-Gly, Ca-Gly-COOH, and Ca-Gly-Maltose BioMOFs decreased from 19.733 to 10.9 m^2^/g and 2.8153 m^2^/g after carboxylation and coating with Maltose (Table 1). To address this issue, the N2 adsorption isotherms and pore size distribution diagrams of the nanoparticles have been shown in Figure 2D. The BJH method was used to determine the pore size distribution, which confirmed the mesoporous character of the nanoparticles.

**TABLE 1:** Overview of the main features of the uncoated and coated BioMOFs.

Features	Ca-Gly	Ca-Gly-COOH	Ca-Gly-Maltose
BET surface Area (m^2^/g)	19.73	10.90	2.81
Mean pore diameter (nm)	15.02	4.82	4.36
Hydrodynamic Size (nm)	111 ± 4.3	122 ± 6.8	181±7.1
ζ-potential (mV)	6.2 ± 4.66	−4.63 ± 3.18	−2.8 ± 0.60

The hydrodynamic size of the Ca-Gly, Ca-Gly-COOH and Ca-Gly-Maltose NPs were 111 nm, 122 and 181 nm, respectively (Table 1). The increase in the hydrodynamic diameter of the NPs after coating is consistent with the existence of carboxylic acid and maltose around the NPs. The ζ-potentials of Ca-Gly, Ca-Gly-COOH and Ca-Gly-Maltose NPs were 6.2 ± 4.66, −4.63 ± 3.18 and −2.8 ± 0.6 mV in PBS solution, respectively (Table 1). Therefore, the presence of carboxylic acid negatively decreased the surface charge of Ca-Gly-COOH, while maltose coating positively increased the surface charge.

Scanning electron microscopy analysis of Ca-Gly, Ca-Gly-COOH, and Ca-Gly-Maltose NPs was also performed. The particle sizes were 60–79 nm, 62–105 nm and 61–126 nm for Ca-Gly, Ca-Gly-COOH and Ca-Gly-Maltose NPs, respectively. SEM analysis shows that the Ca-Gly was spherical, and no morphological changes were observed after coating (Figure 2F).

### Drug Loading Assessment on the Ca-Gly-Maltose BioMOF

At first, the PG and SIM loading on synthesized BioMOFs was performed individually in the concentration range of 50–400 nmol (∼16.17–129.37 μg/mg for PG and 20.93–167.5 μg/mg for SIM). PG was attempted to be loaded up to 64.90 μg/mg with a loading efficiency of 94.72%, and SIM was loaded up to 110 μg/mg with an efficiency of 66.25%. The combined effect of PG and SIM (PG + SIM) on cancer cells was investigated by applying a 1:1 ratio of the drugs (PG IC50/SIM IC50) based on their IC_50_ values. Due to the high sensitivity of the cells to the combination of PG and SIM, the drugs were loaded at a lower ratio (PG:SIM = 1:3.5) for co-loading. Therefore, 3% (w/w) of PG (30 μg/mg) with an efficiency of 98.3 ± 1.24 and 11% (w/w) of SIM (110 μg/mg) with an efficiency of 66.25 ± 2.67% were loaded onto the Ca-Gly-Maltose BioMOF for further investigations. After 4 days of incubation at 4°C, 94.57% of PG and 64.54% of SIM were still present.

### *In vitro* Drug Release From Ca-Gly-Maltose Nanoparticles

In order to simulate the biological condition of lysosome, tumor microenvironment, and blood stream, the drug release profile of PG and SIM loaded BioMOF was investigated *In vitro* and monitored by UV-visible spectrophotometer at 239 nm and 535 nm for PG and SIM, respectively.

**FIGURE 3:**
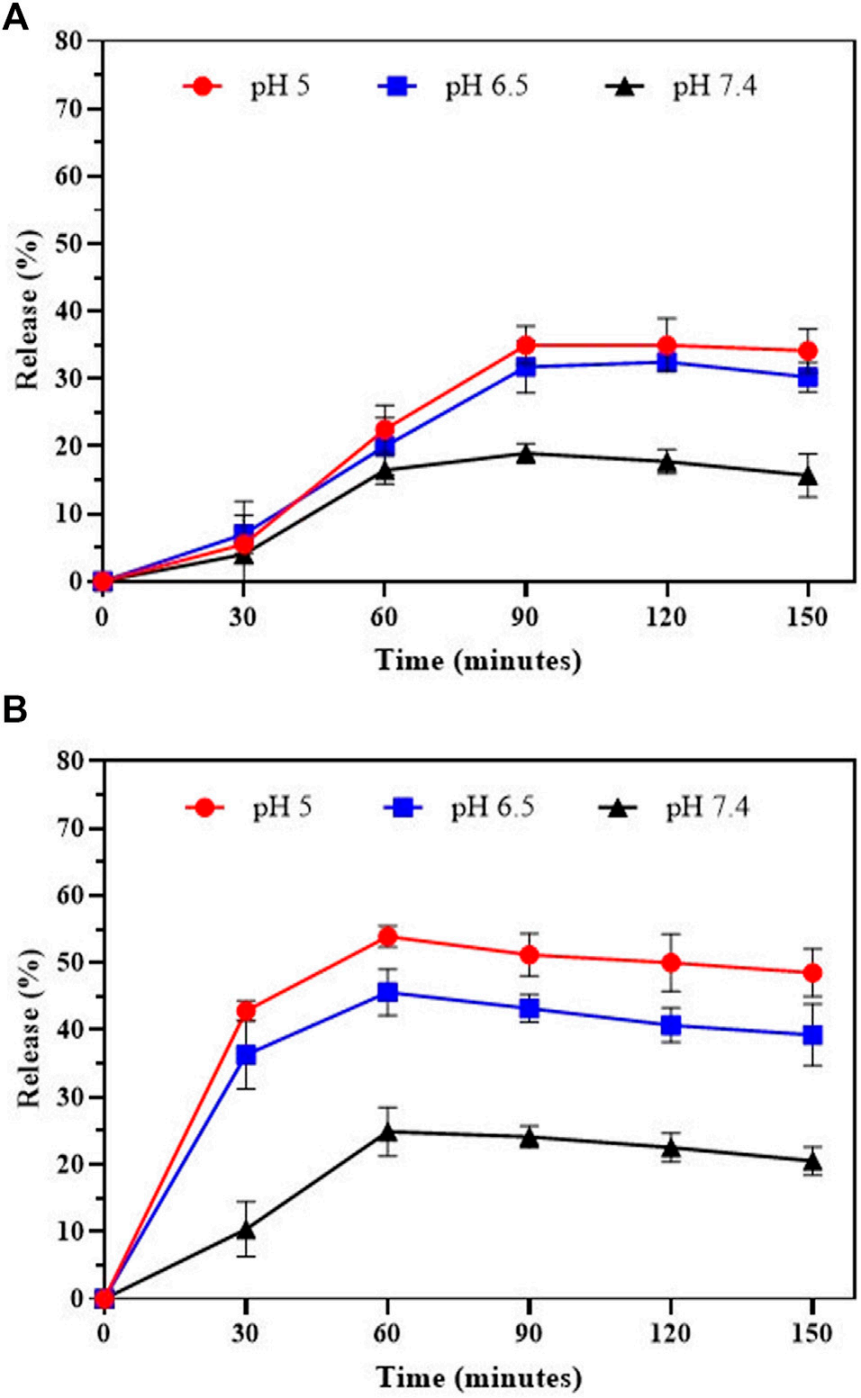
*In-vitro* drug release of **(A)** Prodigiosin and **(B)** Simvastatin from Ca-Gly-Maltose in PBS at pH 5, 6.5 and 7.4.

As shown in Figure 3A, the PG release at physiological pH (∼7.4) was slower than that at pH 6.5 and 5.0, with an initial ∼4% release during 30 min and ∼15.72% release after 150 min. The PG release accelerates up on increasing of acidic conditions, showing ∼5.5 and 7% drug release after 30 min, respectively. However, the release was increased in acidic conditions after 60 min and reached to ∼31% and ∼35% for PG within 90 min at pH 6.5 and 5.0, respectively.

Also, the SIM shown the same pattern of release at neutral pH, with an initial ∼10.38% release during 30 min and 20.5% release in 150 min (Figure 3B). The drug release rate is much faster in acidic conditions, showing ∼45% and ∼54% unloading after 60 min incubation at pH 6.5 and 5.0, respectively. The results showed that the pH- dependent release of PG was much lower than Simvastatin. The drug release profiles for PG and SIM were pH-dependent and time-dependent. In acidic conditions, release of drugs is faster than physiological pH and 6.5. PH-dependent release behavior can be useful for the development of the drug delivery systems in cancer cells due to the acidic pH of the tumor environment. According to reports, drug release from MOFs is pH-dependent (Liu et al., 2019; Xiong et al., 2020; Ma et al., 2021). Rapid release in acidic conditions may be due to protonation of NPs and their structure degradation. With the decrease of the pH values of PBS media, the NPs exhibit an accelerated degradation. Our data also showed that the release rate of Simvastatin was higher than Prodigiosin which is may because of more hydrophobic nature of PG in compare to SIM.

### Cloning, Expression, and Purification of D16F7 scFv

**FIGURE 4:**
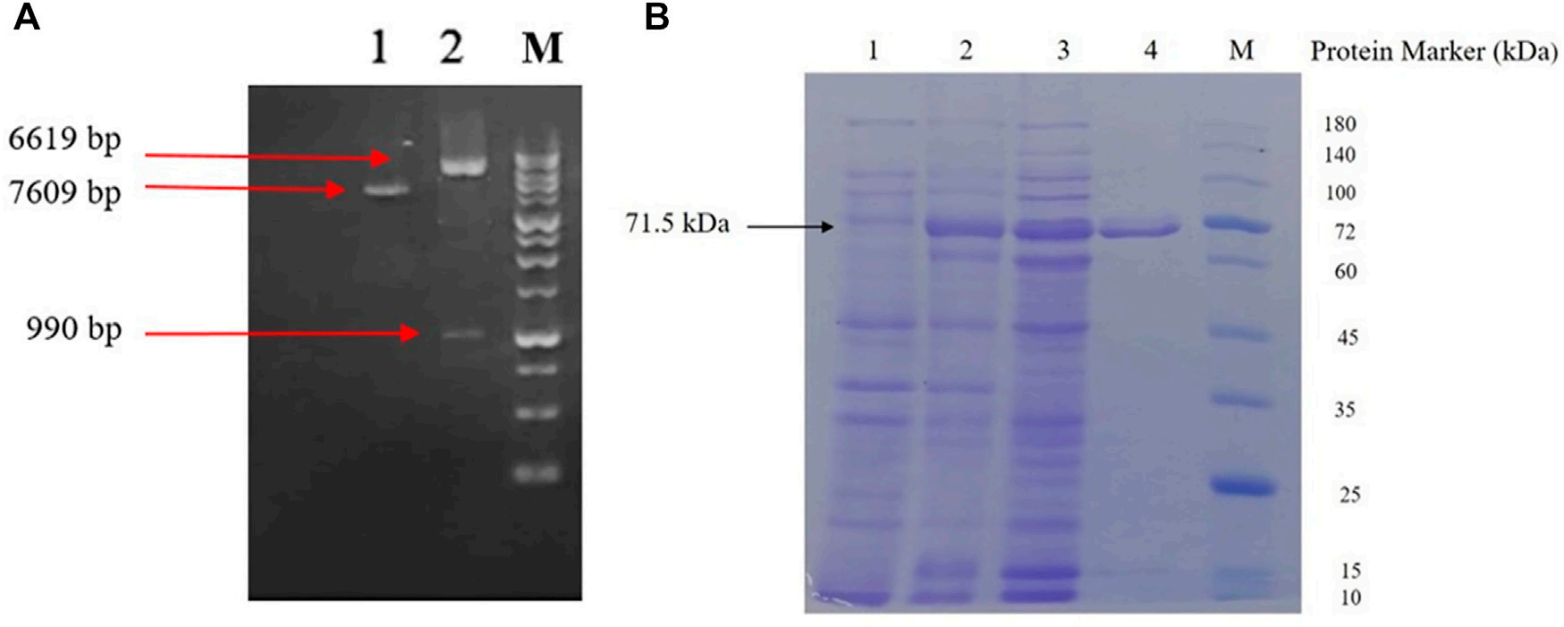
**(A)** Agarose gel electrophoresis analysis; lane 1: undigested pMAL-c2X-D16F7 construct; lane 2: Double-digested pMAL-c2X-D16F7 plasmid showing correct size of plasmid, insert and M: DNA ladder (GeneRuler™ 1 kb). **(B)** SDS-PAGE analysis of recombinant MBP-D16F7 scFv expression; lane 1: un-induced *E. coli* cells; lane 2: cell’s total proteins after auto-induction; lane 3: soluble proteins after auto-induction; lane 4: Ni-purified recombinant fusion protein; and M: protein marker (PM 1500, ExcelBand™).

As shown in Figure 4A, double digestion of the new construct yielded 6,619 and 990 bp DNA fragments, confirming proper integration of the scFv insert in downstream of a maltose binding protein gene sequence followed by a Ser-Asn linker into the pMAL-c2X plasmid (Supplementary Figure S1 for more detail).

Co-expression of the enzymes of the pMJS205 plasmid and the MBP partner protein in pMAL-c2X-D16F7 resulted in a sufficient amount of soluble MBP-scFv in the cytoplasm of *E. coli,* when cultured in ZYM-5052 autoinduction medium for 24 h. As shown in Figure 4B, partial purification of the fusion protein was achieved using Ni-NTA agarose column. A single band with suitable homogeneity and a molecular mass of approximately 71.5 kDa was seen in SDS-PAGE.

### *In Vitro* Cytotoxicity Assessment

The MTT assay was performed to determine the viability of cells after treatment with the different formulations as a function of time and concentration. First, the cytotoxicity of blank nanoparticles (Ca-Gly-Maltose) was investigated in cancerous and normal cell lines. The unloaded synthesized nanoparticles showed very low toxicity in the concentration ranges of 6.25–300 μg/ml, and no significant differences were observed between the negative control and cells exposed to increasing concentrations of the nanoparticles (Supplementary Figure S2A). The purified free MBP-D16F7 protein had very low cytotoxicity in the concentration range of 3.125–200 μg/ml, although the viability of cancer cells decreased significantly compared with normal cell at concentrations above 100 μg/ml (Supplementary Figure S2B). The empty BioMOF-D16F7 showed a slightly stronger toxic effect than the blank nanoparticles and the free MBP-scFv (Supplementary Figure S2C).

The IC_50_ values for free PG and SIM were measured after 24 and 48 h in three different cancer cell lines (MCF-7, LnCap and U87MG) and one non-cancerous cell (Human Skin Fibroblast, HSF). Given the known selectivity of PG for cancer cells (Pérez-Tomás and Vinas, 2010; Rastegari and Karbalaei-Heidari, 2016), the free PG at a range of concentrations (from 0.161 μg/ml to 16.17 μg/ml) and exposure times showed a higher cytotoxicity for the cancer cell lines than for the HSF cells (Supplementary Figure S3). In accordance with these differences, the selective index (SI) values of the free PG on MCF-7, LnCap, and U87MG cell lines were determined as 2.53, 1.94, and 1.90 for 24 h and 2.01, 1.42 and 1.78 for 48 h, respectively. Similarly, SIM also showed an increase in cytotoxicity with increasing of the concentrations (from 0.209 μg/ml to 41.87 μg/ml) and exposure time, although its IC50 values were higher than those of PG (Supplementary Figure S3).

**FIGURE 5:**
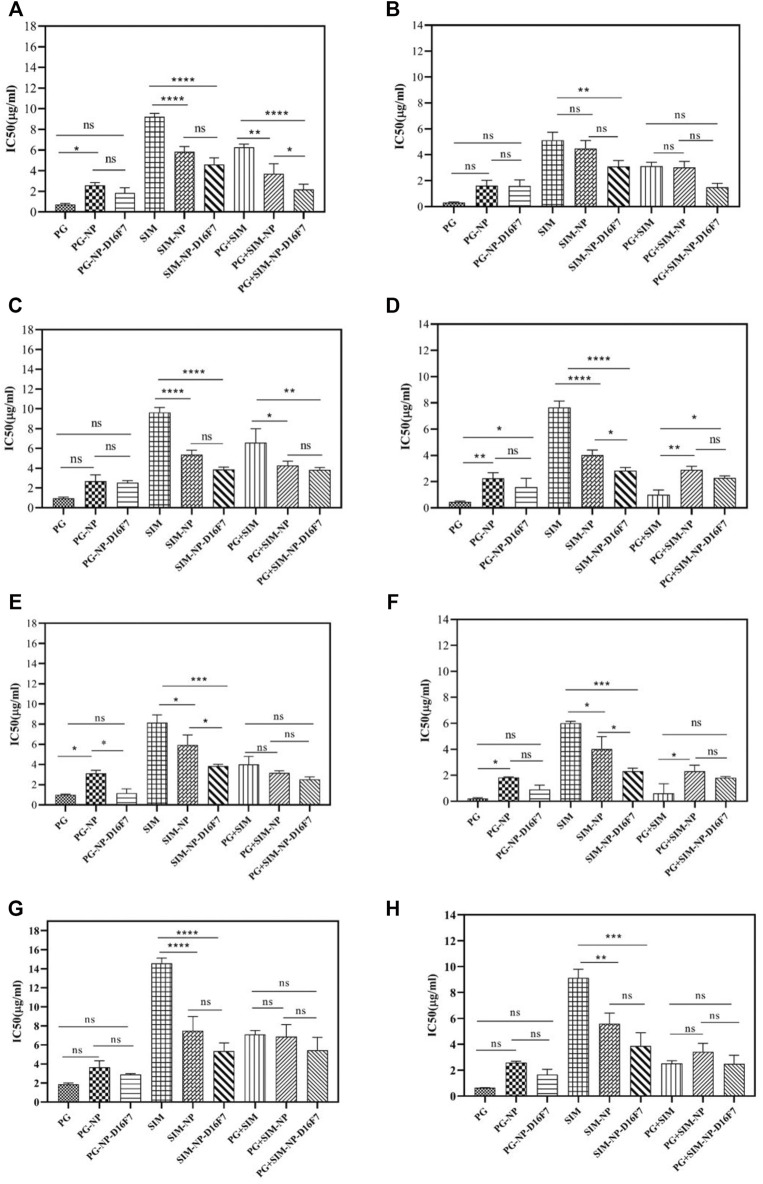
IC_50_ values of free drugs, drug-loaded, and D16F7-functionalized Ca-Gly BioMOFs on MCF-7 cell line after 24 h **(A)** and 48 h **(B)**; on LnCap cells after 24 h **(C)** and 48 h **(D)**; on U87MG cells after 24 h **(E)** and 48 h **(F)**, and on normal HSF cells after 24 h **(G)** and 48 h (H), respectively. Data are mean ± SD. *indicated *p* < 0.05, **indicated *p* < 0.01, ***indicated *p* < 0.001 and *p*< ****indicate 0.0001 (compared with control group).

As shown in Figure 5, the IC_50_ of the PG-NPs was higher than that of free PG at 24 and 48 h, indicating lower toxicity of the compounds after loading, probably due to the slow release of drug from the nanoparticles. However, SIM-NPs showed higher toxicity on cells than the free SIM (lower IC_50_). For SIM-loaded BioMOFs, higher cytotoxicity of drug-loaded NPs and D16F7-functionalized NPs was observed in all tested cell lines.

To have better comparison, we summarized the IC_50_ values of all formulations after 24 and 48 h incubation in different cell lines (Supplementary Table S1). PG-NP-D16F7 and SIM-NP-D16F7 showed significantly improved cytotoxic activity in different cell lines after 24 and 48 h treatment compared with non-targeted nanoparticles. However, the highest cytotoxicity for both PG-NP-D16F7 and SIM-NP-D16F7 was in U87MG cells, with IC_50_ values of 1.17 and 3.86 μg/ml after 24 h and 0.91 and 2.31 μg/ml after 48 h, respectively.

These results are consistent with the aim of the study to improve drug cytotoxicity on cancerous cells after loading and functionalization of Ca-Gly BioMOFs. Statistical analysis revealed that although there were no significant differences between the PG-loaded NPs and PG-D16F7-BioMOFs on most cell lines, but there were statistically significant effects for SIM-loaded BioMOFs (Figure 5). Overall, incubation of cells with the free drugs and drug-loaded nanoparticles showed that cytotoxicity exhibited a concentration and time-dependent behavior showing an enhancement of toxicity with increasing drug concentration and exposure time, and in some cases when the BioMOFs were functionalized with an anti-VEGFR1 antibody.

### Evaluation of the Combinatorial Effect of Nanoparticles Loaded With Two Drugs

The combined cytotoxic effect of PG and SIM was evaluated by the MTT cytotoxicity assay at an equipotent molar ratio ([PG] at IC_50_ [SIM] at IC_50_). The combination index (CI) has been used to evaluate the synergistic, antagonistic or additive effects of drugs combination. CI > 1 indicates antagonism, CI < 1 indicates synergy and CI = 1 indicates an additive effect (Chou and Martin, 2007; Chou, 2010). The CI of each drug combination was plotted as a function of fractional inhibition (Fa) by computer simulation from Fa = 0.10 to 0.95 (Supplementary Figure S4). As summarized in Table 2, the combination of PG and SIM (PG + SIM) had a greater anticancer effect in their equipotency ratio and showed a lower value of the IC_50_ than the free drugs alone. For example, after 24 h incubation on MCF-7 cells, the PG + SIM combination showed an IC_50_ = 5.99 μg/ml (0.33 μg/ml PG+ 5.66 μg/ml SIM), while the IC_50_ values for the free PG and free SIM were 0.73l and 9.22 μg/ml, respectively (Figure 5; Supplementary Table S1). Table 2 shows the CI values with their interpretations in different cells for PG + SIM and PG + SIM-NP-D16F7 treatment at 24 and 48 h. This result indicates that the PG + SIM inhibits cancer cells growth more effectively than free PG and SIM. Moreover, the CI values obtained for PG + SIM-NP-D16F7 after 24 and 48 h treatment showed synergistic cytotoxicity (Table 2).

**TABLE 2:** Comparison of CI values and their CI interpretation for the combination of free PG + SIM and the PG + SIM-NPs after 24 and 48 h incubation on different cell lines.

Cell Lines	Time Incubation	PG+SIM			PG+SIM-NP-D16F7		
		CI	IC50	Interpretation	CI	IC50	Interpretation
MCF-7	24 h	1.83	5.99 µg/ml (0.33 + 5.66 µg/ml)	antagonism	0.329	2.18 µg/ml (0.0.5 + 1.68 µg/ml)	synergism
	48 h	0.98	3.11 µg/ml (0.17+2.94 µg/ml)	Synergism	0.197	1.49 µg/ml (0.0.349 + 1.14 µg/ml)	synergism
LnCap	24 h	0.68	6.58 µg/ml (0.47+6.11 µg/ml)	Synergism	0.34	3.83 µg/ml (0.891 + 2.94 µg/ml)	synergism
	48 h	0.34	2.99 µg/ml (2.07+0.92 µg/ml)	Synergism	0.196	2.29 µg/ml (0.533 + 1.76 µg/ml)	synergism
U87MG	24 h	0.414	4.0 µg/ml (0.49+3.51 µg/ml)	Synergism	0.328	2.514 µg/ml (0.584 + 1.93 µg/ml)	synergism
	48 h	0.001	0.61 µg/ml (0.08+0.53 µg/ml)	Synergism	0.19	1.80 µg/ml (0.418 + 1.38 µg/ml)	synergism
HSF	24 h	1.58	7.10 µg/ml (0.56+6.54 µg/ml)	antagonism	0.434	5.43 µg/ml (1.26 + 4.17 µg/ml)	synergism
	48 h	0.575	3.84 µg/ml (0.274+3.56 µg/ml)	Synergism	0.294	2.49 µg/ml (0.58 + 1.91 µg/ml)	synergism

The CI value of PG + SIM was less than PG + SIM-NP-D16F7 after 48 h in U86MG cells. Cells are more sensitive to free drugs than drug-loaded nanoparticles where have slower release. The PG + SIM-NPs-D16F7 showed lower IC_50_ values than the free PG + SIM and PG + SIM-NPs which could be attributed to the functionalization of the nanoplatform with the D16F7 scFv, confirming the effect of targeting on the VEGFR1 receptor on cancer cells, although no significant differences were seen between them except for MCF-7 (Figure 5). Easy accessibility of cells for the hydrophobic drugs is the main reason of these observations which obscures the targeting behavior of antibodies in Ca-Gly-Maltose-D16F7.

### *In Vitro* Cellular Uptake

The efficiency of cellular uptake was assessed using an inverted fluorescence microscope. The results showed that these formulations were able to deliver the drugs into cells, and the fluorescence increased dramatically after 90 min (Supplementary Figure S5). Since the PG has autofluorescence, its penetration into cells can be assessed with a fluorescence microscope. The hydrophobicity of the compounds contributes to their ease of penetration into the cell in free form. However, the higher fluorescence emission of cells treated with PG + SIM-NP-D16F7 compared with PG-SIM-NP in a short time (30 min) after addition showed that the targeting design worked well and uptake of the smart nanocarrier occurred *via* antibody-receptor interactions (Supplementary Figure S5).

### *In Vitro* Scratch Assay

**FIGURE 6:**
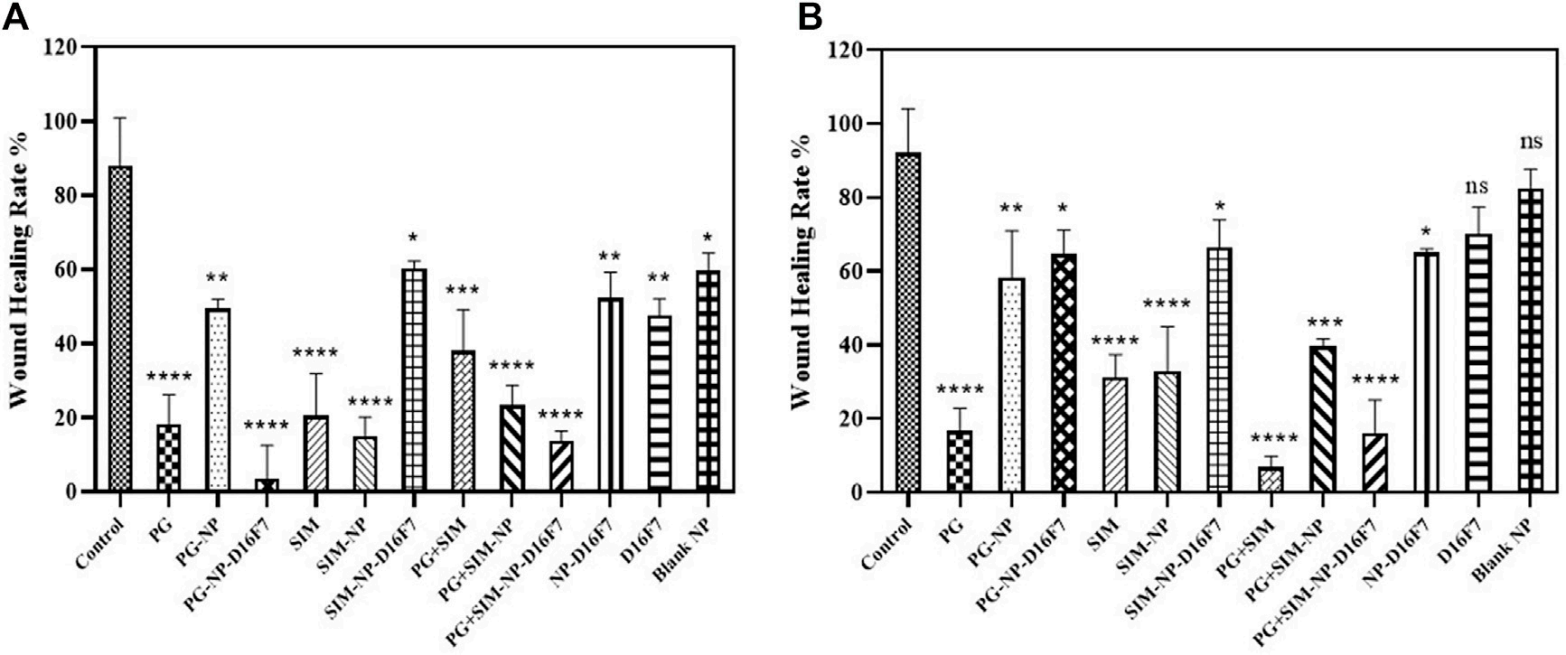
Comparative evaluation of the percentage of wound healing of different drug formulations at 24 h **(A)** and 48 h **(B)** on MCF-7 cell line. Significance was defined for *p* < 0.05.

Cellular migration or tumor invasion is a crucial phenomenon in carcinogenesis, as metastasis of tumor cells occurs in this way. According to the MTT results, the developed nanoplatforms significantly decreased the cell viability of MCF-7 cell line. In our study, a scratch assay was performed at IC_20_ concentrations to examine the potential of the free drugs and the developed drug-loaded nanocarriers to inhibit cell migration. Since the cells will more rapidly die at IC_50_ of the drugs, for wound healing assay, a sub-toxic (IC_20_) concentration is generally used to perform the experiment. As shown in Figure 6, the cell migration inhibition assay revealed a reduction in cellular proliferation and migration when treated with the various formulations compared to the control group (without treatment).

For the free PG and SIM at IC_20_ concentration, there was a significant difference in cell migration (18.32% at 24 h and 16,79% at 48 h for the free PG and 20.51 and 31.28% at 24 and 48 h, for the free SIM); while the wound healing rate was 38.04 and 6.82% at 24 and 48 h for the combination of free PG and SIM. The PG-NP, the SIM-NP, and the PG + SIM-NP were also able to inhibit the cell proliferation weaklier than the free PG, SIM and the PG + SIM, probably due to the slow penetration of the nanoparticles. Inhibition of cell migration by PG-NP-D16F7 and PG + SIM-NP-D16F7 was significantly higher than that of PG-NP and the PG + SIM-NP, respectively; while there was no significant difference between SIM-NP-D16F7 and SIM-NP. The D16F7 scFv and functionalized nanoparticles (D16F7-NP) showed a reduction in cell migration compared to the blank nanoparticles and untreated control, highlighting the superiority of D16F7 for targeting cancer cells.

## Discussion

Nanotechnology-based carriers for single or multiple drugs are expected to improve the efficiency of treatments and reduce the toxic effects of chemotherapeutic drugs on normal cells (Martinelli et al., 2019; Yan et al., 2020). The synthesis and preclinical studies of various nanoparticles are being intensively under investigation to achieve appropriate targeted therapy with longer survival and better patient well-being (Mekonnen et al., 2019; Mura, 2020). Although it has been reported that various nanocarrier-based drug delivery systems provide ideal chemotherapy (Aryal et al., 2010; Wang et al., 2011; Hossen et al., 2019; Kucuksayan et al., 2021), the process still needs to be improved. Anticancer drug delivery or co-administration with receptor-guided BioMOFs that are safe, efficient, and smart was the main goal of the present project. Targeted co-delivery of Prodigiosin (PG) and simvastatin (SIM) *via* a newly synthesized BioMOF coated with the maltose and functionalized with a fusion MBP- D16F7 scFv was introduced to achieve a smart targeting and enhance the synergistic effect of an anti-cancer drug candidate and a cholesterol reducing agent.

In the present work, we have developed for the first time a new mesoporous BioMOF composed of biocompatible and bioactive Ca^+2^ metal ions and a biological linker, glycine as a biological drug delivery system. The nanoparticles with a size range of 70–150 nm can be taken up into cells by clathrin-mediated endocytosis (Manzanares and Ceña, 2020). In this study, the size of nanoparticles ranged from 60 to 126 nm. The zeta potential values of the Ca-Gly, Ca-Gly-COOH and Ca-Gly-Maltose NPs were 6.2 ± 4.66, −4.63 ± 3.18 and −2.8 ± 0.6 mV, respectively. Coating the carboxylated BioMOF with maltose facilitated the internalization process by reducing the negative potential of the nanoparticles surface. Karimi et al. reported that the use of maltose as a capping agent in the synthesis of Fe_3_O_4_@C@TDGQDs microsphere resulted in a positive surface charge and could be internalized into the cells due to the negative cell surface (Asati et al., 2010; Karimi and Namazi, 2020). The morphology of the synthesized nanoparticles is spherical. Several studies have demonstrated that spherical NPs undergo higher cellular uptake than rod-shaped NPs, because membrane wrapping for rod-shaped NPs takes longer time than for spherical NPs (Behzadi et al., 2017). Moreover, hydroxyl groups on maltose may improve the uptake of synthesized BioMOF into the cells via hydrogen bonds (Pereira and Hunenberger, 2006).

Focusing on an actively targeted drug delivery system based on cell-specific ligands can enhance the effects of passive targeting and improve pathway of drug targeting to a specific site (Biffi et al., 2019; Lü et al., 2021). The VEGFR-1 receptor is overexpressed by many cancers, which promotes cell proliferation, tumor progression, angiogenesis, and metastasis (Wey et al., 2005). Activation of the VEGFR-1 signaling pathway promotes tumor vascularization and cell growth, and inhibits apoptosis (Wu et al., 2006a; Wu et al., 2006b). Graziani et al. developed an anti-VEGFR-1 mAb (D16F7) by immunizing BALB/C mice which markedly inhibited angiogenesis, endothelial cell migration and intracellular signal transduction in melanoma and glioblastoma (Graziani et al., 2016; Atzori et al., 2017).

Based on the above information, we developed a BioMOF functionalized with a recombinant anti-VEGFR1 scFv to specifically deliver the PG and SIM to VEGFR-1 expressing cancer cells. A co-expression system was used to produce a soluble fusion MBP-D16F7 scFv in *E. coli.* The scFv fragment was developed as a fusion protein MBP-scFv, which not only provides a more soluble and functional protein, but also can bind the scFv to the maltose on the surface of BioMOFs (Ca-Gly-Maltose) (Reche-Perez et al., 2021). Our results showed that the synthesized BioMOFs were safe and nontoxic as empty carriers and that the unloaded Ca-Gly-D16F7 BioMOF had a stronger effect on cancer cells than on normal cells by inhibiting the proliferation of eukaryotic cells especially at a concentration greater than 100 μg/ml (Supplementary Figure S2).

The goal of co-delivery systems is to combine two or more drugs with different properties and mechanisms, which in turn can improve therapeutic effects and/or reduce their adverse effects in cancer therapy (Meng et al., 2020; Wang et al., 2021). In recent years, many efforts have been made to overcome MDR such as P-glycoprotein (P-gp) through various drug strategies (Sharom, 2008). Cholesterol and sphingolipids are essential components of membrane microdomains known as rafts. ABC transporters such as P-gp are thought to be associated with lipid rafts (Klappe et al., 2009). By lowering cholesterol and sphingolipids levels, SIM inhibits the transport activity of ABC transporters, such as P-gp. Goard et al. showed that lovastatin binds directly to P-gp and thus affects Dox transport in cancer cells (Goard et al., 2010). In the present study, the co-delivery of SIM and PG based on a targeting nanocarrier platform was considered and investigated on cell line culture systems. The PG compound was introduced as a proapoptotic ligand via cleavage of double stranded DNA and disruption of the pH gradient (Pérez-Tomás and Vinas, 2010; Anwar et al., 2020). According to our results, the present co-drug delivery formulation showed a stronger cytotoxic effect than the nanoparticles loaded with only one drug (PG or SIM) or the free drugs. This synergistic effect can be explained by the fact that PG targets the MAPK/mTOR/p70S6K growth pathway and SIM targets the apoptotic Bax/Bcl-2 pathway (Campbell et al., 2006; Spampanato et al., 2012). As shown in Table 2, and based on the calculated CI values, synergistic inhibitory effects on cell viability of cancer cells were observed for the free PG + SIM and the PG + SIM-NP-D16F7 (Supplementary Figure S3; Supplementary Table S1). Jamil et al. demonstrated that the combination loaded nanoparticles with Gemcitabine and Simvastatin exhibited higher intracellular uptake and cytotoxicity to pancreatic cancer cells, while being safer for normal cells (Jamil et al., 2019). Our results showed that the inhibitory effect of PG + SIM-NP-D16F7 increased compared with PG + SIM-NP and PG + SIM which can be attributed to the presence of scFv D16F7 on the surface of NPs (Figure 5). The internalization of the targeted delivery system into cancer cells and the release of the cytotoxic agents are also appropriate (Supplementary Figure S5). In the present *in vitro* model (cell line toxicity assessment), the targeted nanoparticles showed improved cytotoxic activity compared to non-targeted nanoparticles and free combined drugs; however, these are not significant except for the MCF-7 cell line (Figure 5). The main reason for these observations is the easy accessibility of the cells to the free drugs, which obscures the actual targeting behavior of the antibody-receptor system in Ca-Gly-Maltose-D16F7. Therefore, the *in vivo* animal model assays are suggested that can survey the internalization and cytotoxicity of the developed nanocarrier in detail.

Another important feature studied was cell migration after various treatment using a scratch assay in a cell culture model. Although the migration assay showed that the combination of PG and SIM inhibited cell migration, PG + SIM-NP-D16F7 treatment was more effective than PG + SIM-NP in the migration inhibition test. The results also demonstrate apoptosis features such as the fragmented nuclear DNA through DAPI staining (Supplementary Figure S5). The produced nanocarriers inhibit cell growth and induce apoptosis, but the extent of apoptosis cannot be detected by flow cytometry because of the emission wavelength of prodigiosin which was similar to that of propidium iodide. Thus, the combination of the PG and SIM targeting VEGFR-1 overexpressed in cancer cells can be introduced as a potent dual drug administration. Further studies to evaluate the *in vivo* efficacy of the developed nanocarriers are proposed to investigate the degree of therapeutic effects and overcoming MDR in malignant tumors in preclinical research.

## Conclusion

A newly synthesized Ca-Gly-Maltose nanoparticles with suitable physiochemical characteristics was introduced. The Ca-Gly-Maltose functionalized with recombinant anti-VEGFR1 scFv, was used to load PG and SIM, either alone or in combination, and its therapeutic effect was investigated by cytotoxicity evaluation in a cell culture model. The mesoporous BioMOFs represent an effective nanocarrier that can accommodate multiple drugs and can be functionalized with various scFv fused to the maltose binding protein. The nanoparticles coated with anti-VEGFR1 antibodies showed good loading capacity for the drug and were still able to target the corresponding receptor. Combination therapy with PG + SIM in nanoparticles conjugated with anti-VEGFR1 scFv improved their permeability and cytotoxicity on cells overexpressing VEGFR1 receptor. The smart drug delivery system provided in our study may be a promising candidate for the treatment of cancer while further studies on the stability of NPs and evaluation of cytotoxicity in animal models are suggested.

## Data Availability

The original contributions presented in the study are included in the article/Supplementary Material, further inquiries can be directed to the corresponding authors.
