# Supplementary material for: Targeted Codelivery of Prodigiosin and Simvastatin Using Smart BioMOF: Functionalization by Recombinant Anti-VEGFR1 scFv
Source: Front Bioeng Biotechnol. 2022 Mar 24;10:866275. doi: 10.3389/fbioe.2022.866275 (PMC8987009; doi:10.3389/fbioe.2022.866275)
Supplement: Supplementary file 1 [file DataSheet1.PDF]

Supplementary Information

Targeted Codelivery of Prodigiosin and Simvastatin Using Smart BioMOF: Functionalization by Recombinant Anti-VEGFR1 scFv

Somayyeh Mirzaeinia[1], Sedighe Zeinali[2], Nediljko Budisa[3,4,*‡], and Hamid Reza Karbalaei-Heidari[1,3,*‡]

[1] Molecular Biotechnology Lab, Department of Biology, Faculty of Science, Shiraz University, Shiraz, Iran.

[2] Department of Nanochemical Engineering, School of Advanced Technologies, Nanotechnology Research Institute, Shiraz University, Shiraz, Iran.

[3] Department of Chemistry, Faculty of Science, University of Manitoba, Winnipeg, MB., Canada. R3T 2N2

[4] Institut für Chemie, Technische Universität Berlin, Müller-Breslau-Straße 10, 10623 Berlin, Germany.

***Correspondence**:

Nediljko Budisa, nediljko.budisa@tu-berlin.de; nediljko.budisa@umanitoba.ca

Hamid Reza Karbalaei-Heidari, karbalaei@shirazu.ac.ir; Hamid.karbalaeiheidari@umanitoba.ca

[‡]These authors have contributed equally to this work and share last authorship

Table S1. Summary list of IC_{50} values for various formulations after 24 and 48 h incubation on different cell lines. * Indicates amount of drug loaded onto the BioMOFs.

Cell line	Incubation time	IC50								
		**PG (μg/ml)**	**PG-NP (μg/ml)***	**PG-NP-D16F7 (μg/ml)***	**SIM (μg/ml)**	**SIM-NP (μg/ml)***	**SIM-NP-D16F7 (μg/ml)***	**PG-SIM (μg/ml)**	**PG-SIM-NP (μg/ml)***	**PG-SIM-NP-D16F7 (μg/ml)***
**MCF-7**	**24 h**	**0.73±0.113**	**2.58±0.262**	**1.85±0.504**	**9.22±0.321**	**5.85±0.5**	**4.6±0.723**	**5.99±0.29**	**3.70±1.96**	**2.18±0.51**
	**48 h**	**0.316±0.038**	**1.62±0.403**	**1.59±0.462**	**5.11±0.622**	**4.48±0.628**	**3.1±0.5**	**3.11±0.293**	**2.46±0.47**	**1.49±0.3**
**LnCap**	**24 h**	**0.950±0.118**	**2.68±0.650**	**2.54±0.211**	**9.63±0.497**	**5.36±0.461**	**3.86±0.254**	**6.58±1.4**	**4.29±0.424**	**3.83±1.21**
	**48 h**	**0.45±0.069**	**2.26±0.410**	**1.57±0.686**	**7.64±0.492**	**4.03±0.374**	**2.84±0.24**	**2.99±0.36**	**2.88±0.282**	**2.29±0.148**
**U87MG**	**24 h**	**0.97±0.078**	**3.13±0.294**	**1.17±0.421**	**8.13±0.805**	**5.94±1.1**	**3.86±0.14**	**4±0.794**	**3.17±0.202**	**2.514±0.258**
	**48 h**	**0.21±0.055**	**1.83±0.04**	**0.91±0.34**	**6.01±0.141**	**4.02±0.97**	**2.31±0.24**	**0.61±0.733**	**2.31±0.463**	**1.80±0.105**
**HSF**	**24 h**	**1.85±0.163**	**3.64±0.7**	**2.88±0.11**	**14.56±0.57**	**7.48±1.51**	**5.34±0.866**	**7.10±0.424**	**6.89±1.24**	**5.43±1.37**
	**48 h**	**0.63±0.003**	**2.59±0.091**	**1.65±0.4**	**9.12±0.67**	**5.59±0.82**	**3.87±1.02**	**3.84±0.212**	**3.43±0.640**	**2.49±0.66**

Figure. S1. Schematic representation of the construct and expression vectors used in this study. A) Structure of pUC-57- D16F7 scFv which was synthesized by Genscript. B) Design of the D16F7 scFv gene sequence, consisting of VH and VL nucleotide sequences joined by a flexible peptide linker sequence [$(G_4S)_3$] and a C-terminal 6 × His tag. **C).** Schematic map of the pMal-c2X-D16F7 scFv construct used to express MBP-D16F7 scFv in *E. coli*. D) Nucleotide sequence of the variable heavy (VH) domain, flexible linker, and variable light (VL) domain of D16F7 scFv.

A)

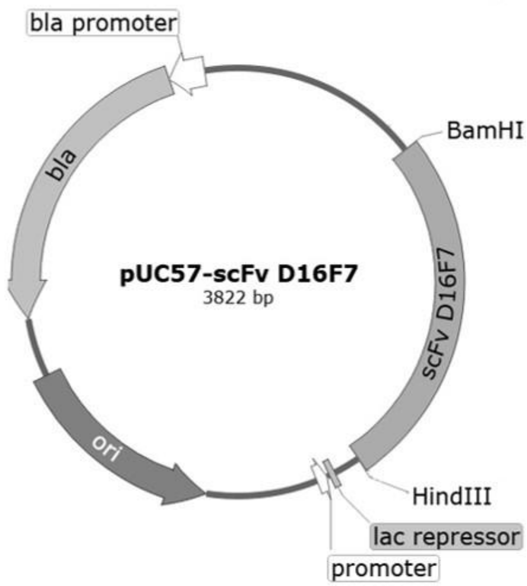

B)

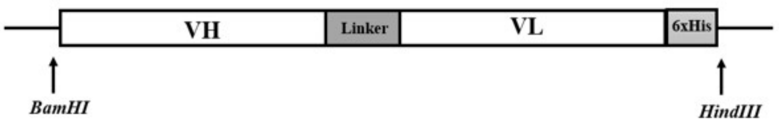

C)

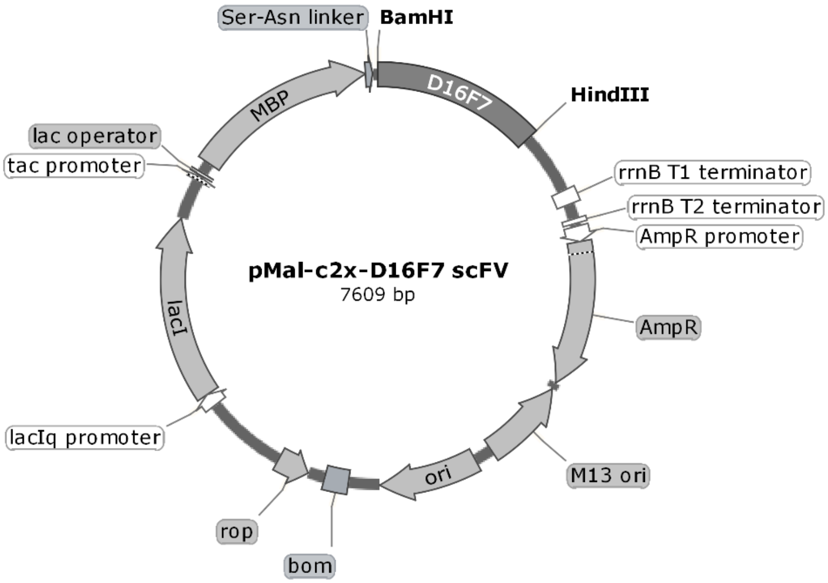

D)

Variable Heavy (VH) Domain:

GATGTGCTGATGACTCAGACCCCTCTGTCTTTGCCGGTGAGCCTGGGCGATCAGGCG
TCTATTAGTTGCCGCTCTAGCCAGAGCATTGTGCATTCCAACGGCAACACCTATCTG
GAATGGTTTTTGCAGAAGCCAGGCCAAAGCCCGAAACTTCTGATCTATAAAGTGAG
CAACCGCTTTAGCGGTATTCCGGATCGCTTTAGCGGCAGCGGTAGCGGCACCGATTT
TACCCTGAAAATTTCTCGTGTGGAGGCGGAAGACCTGGGTGTTTATTTTTGCTTTCAG
GGCAGCCATGTGCCGTATACCTTTGGTGGCGGCACCAAACTGGAAATTAAA

Nucleotide Sequence of Linker:

GGCGGTGGCGGTAGCGGTGGTGGCGGTAGCGGTGGCGGCGGTAGC

Variable Light (VL) Domain:

GATGTGCTGATGACTCAGACCCCTCTGTCTTTGCCGGTGAGCCTGGGCGATCAGGCG
TCTATTAGTTGCCGCTCTAGCCAGAGCATTGTGCATTCCAACGGCAACACCTATCTG
GAATGGTTTTTGCAGAAGCCAGGCCAAAGCCCGAAACTTCTGATCTATAAAGTGAG
CAACCGCTTTAGCGGTATTCCGGATCGCTTTAGCGGCAGCGGTAGCGGCACCGATTT
TACCCTGAAAATTTCTCGTGTGGAGGCGGAAGACCTGGGTGTTTATTTTTGCTTTCAG
GGCAGCCATGTGCCGTATACCTTTGGTGGCGGCACCAAACTGGAAATTAAA

Figure. S2. Cytotoxicity effect of A) the blank BioMOF, B) the free MBP-D16F7 scFv, and C) the unloaded Ca-Gly-D16F7 after 24 and 48 h incubation on the cancer cells (U87mg, LnCap, MCF-7) and normal cell (HSF). Data represent mean ± SD.

A)

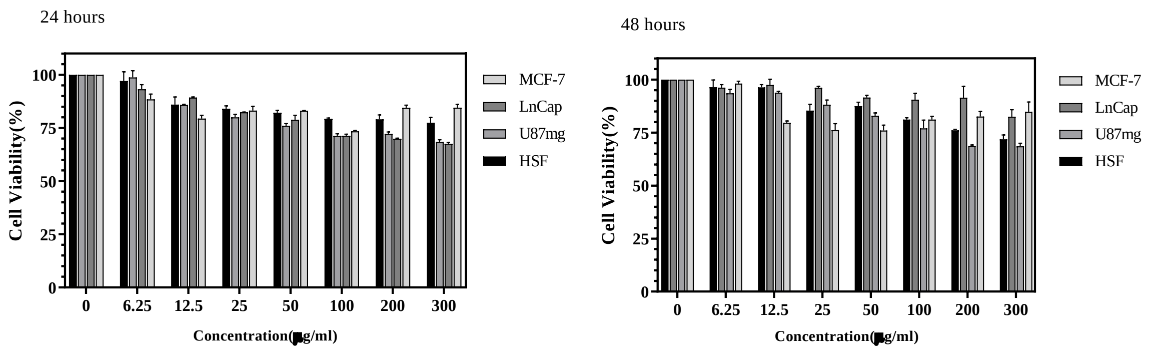

B)

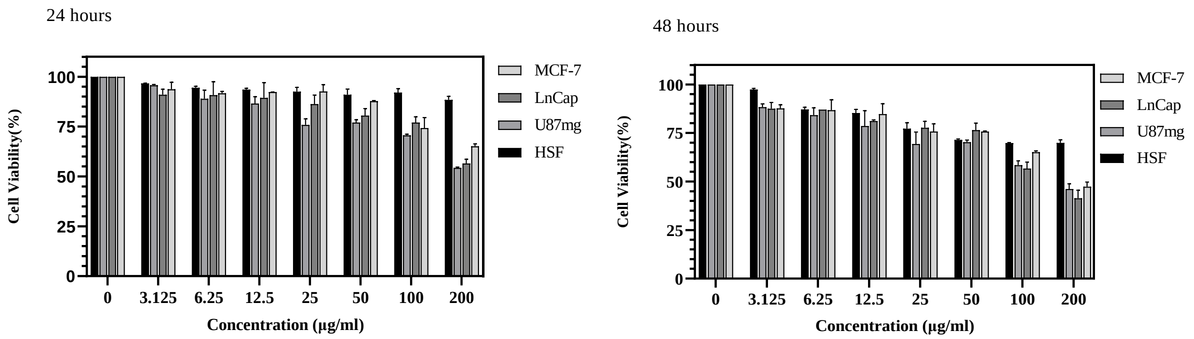

C)

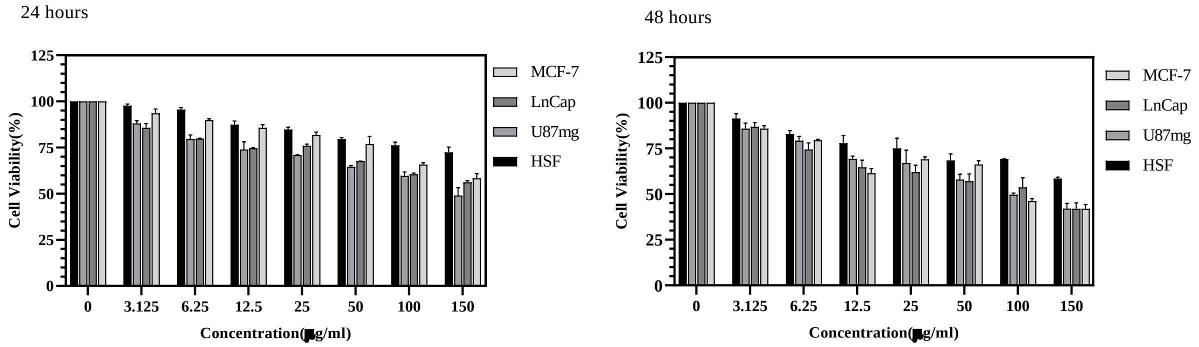

Figure. S3. Percentage of cell viability of various cell lines after 24 h and 48 h incubation by A) the PG, B) the SIM, C) the PG-NP, D) the PG-NP-D16F7, E) the SIM-NP, F) the SIM-NP-D16F7, G) the PG+SIM-NP, and H) the PG+SIM-NP-D16F7 on various cell lines after 24 h and 48 h incubation. The data are mean values ± SD.

A)

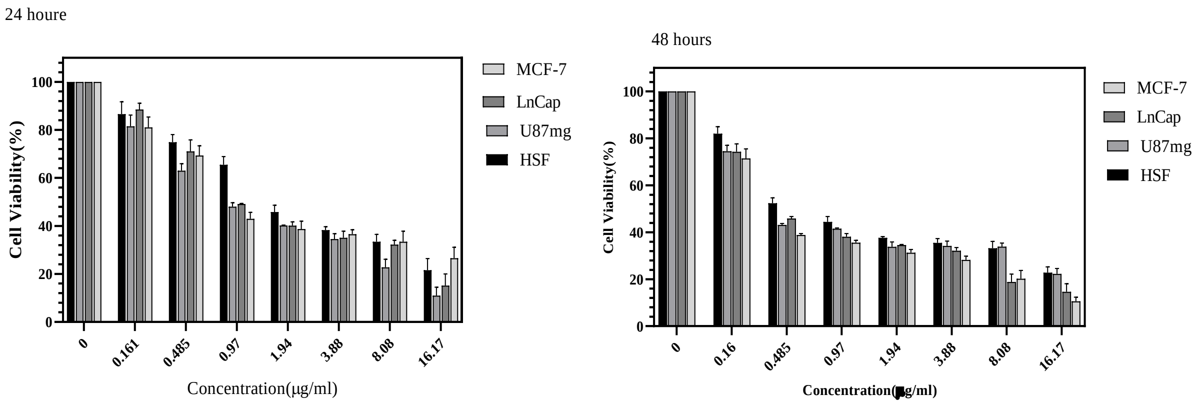

B)

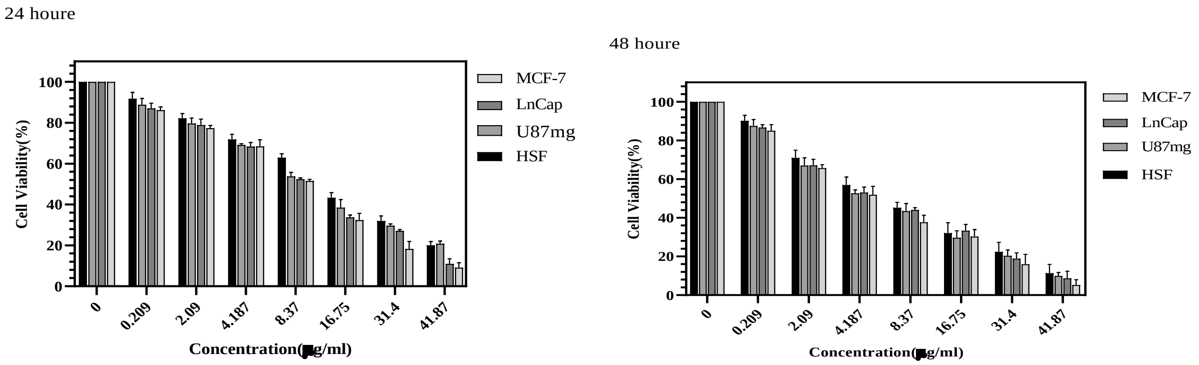

C)

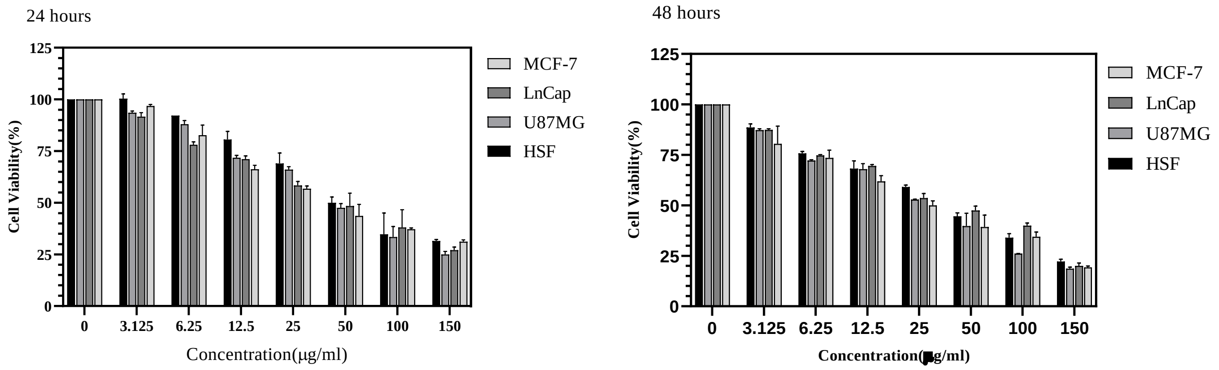
24 hours
48 hours
Cell Viability(%)
Concentration(μg/ml)
MCF-7
LnCap
U87MG
HSF
0
3.125
6.25
12.5
25
50
100
150

D)

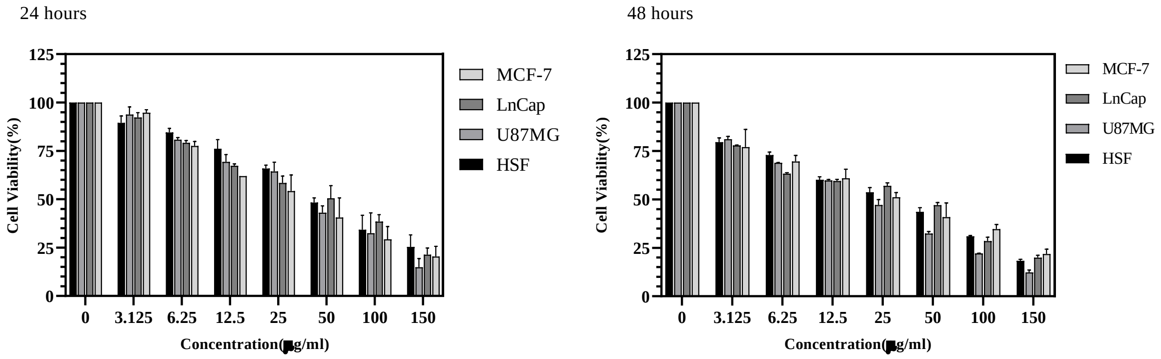
24 hours
48 hours
Cell Viability(%)
Concentration(μg/ml)
MCF-7
LnCap
U87MG
HSF
0
3.125
6.25
12.5
25
50
100
150

E)

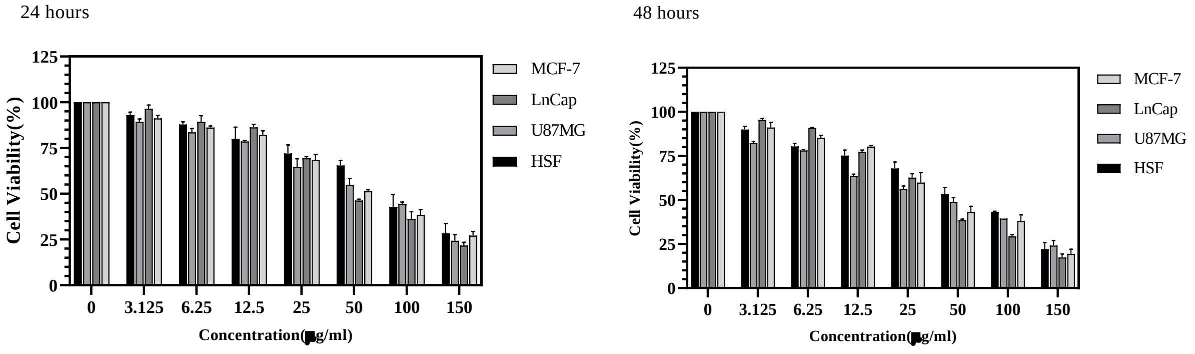
24 hours
48 hours
Cell Viability(%)
Concentration(μg/ml)
MCF-7
LnCap
U87MG
HSF
0
3.125
6.25
12.5
25
50
100
150

F)

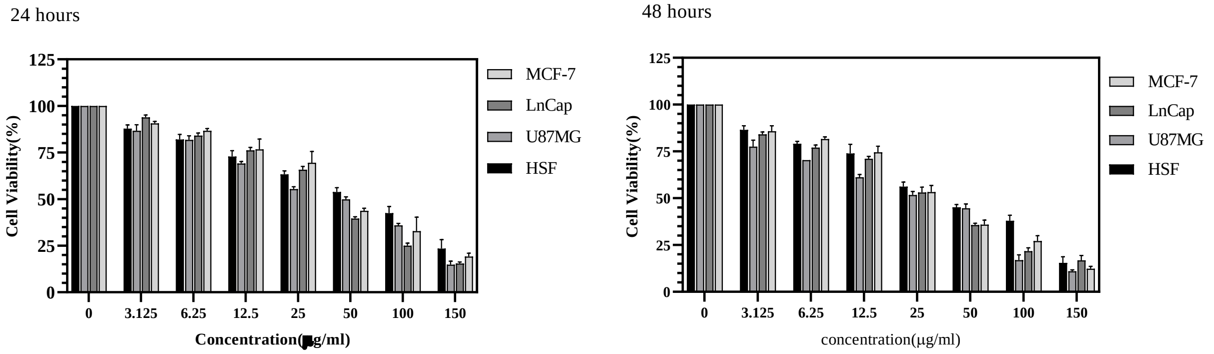

24 hours
48 hours
Cell Viability(%)
125
100
75
50
25
0
0
3.125
6.25
12.5
25
50
100
150
concentration(µg/ml)
MCF-7
LnCap
U87MG
HSF

G)

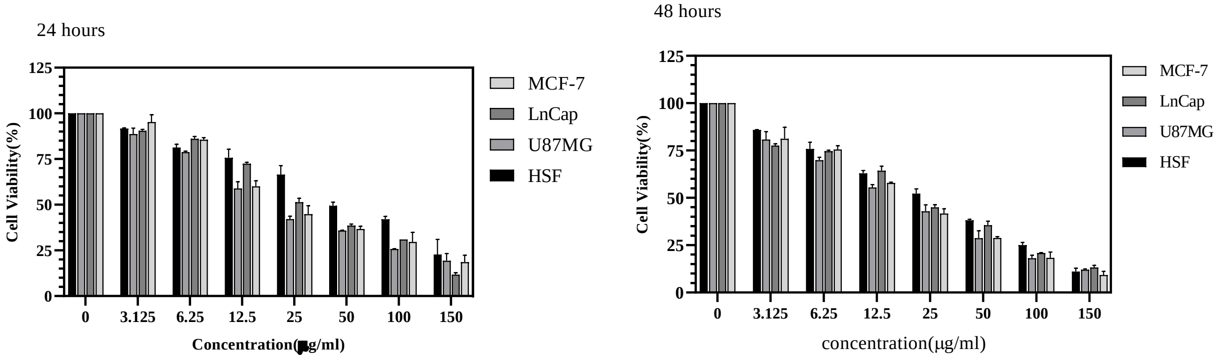

24 hours
48 hours
Cell Viability(%)
125
100
75
50
25
0
0
3.125
6.25
12.5
25
50
100
150
concentration(µg/ml)
MCF-7
LnCap
U87MG
HSF

H)

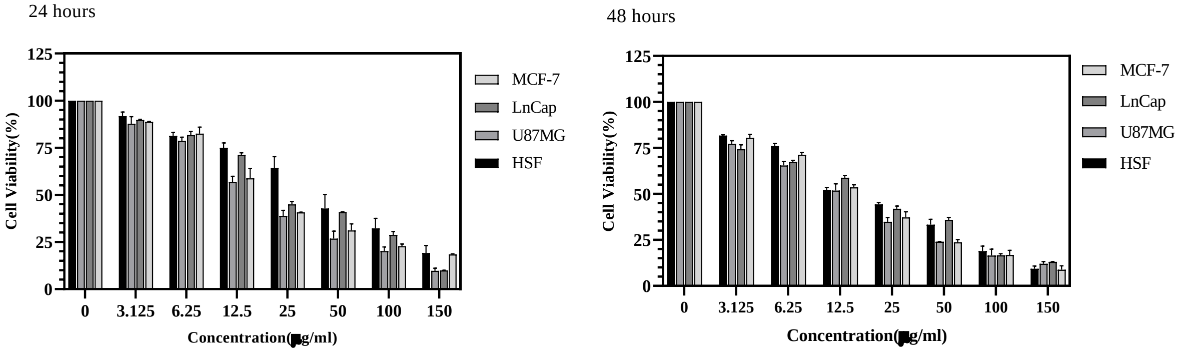

24 hours
48 hours
Cell Viability(%)
125
100
75
50
25
0
0
3.125
6.25
12.5
25
50
100
150
MCF-7
LnCap
U87MG
HSF

Figure. S4. The combination index (CI) plot of PG+SIM and PG+SIM-NP after 24 and 48 h incubation on A) MCF-7, B) LnCap, C) U87MG, and D) HSF cell lines. Data represent the mean ± SD.

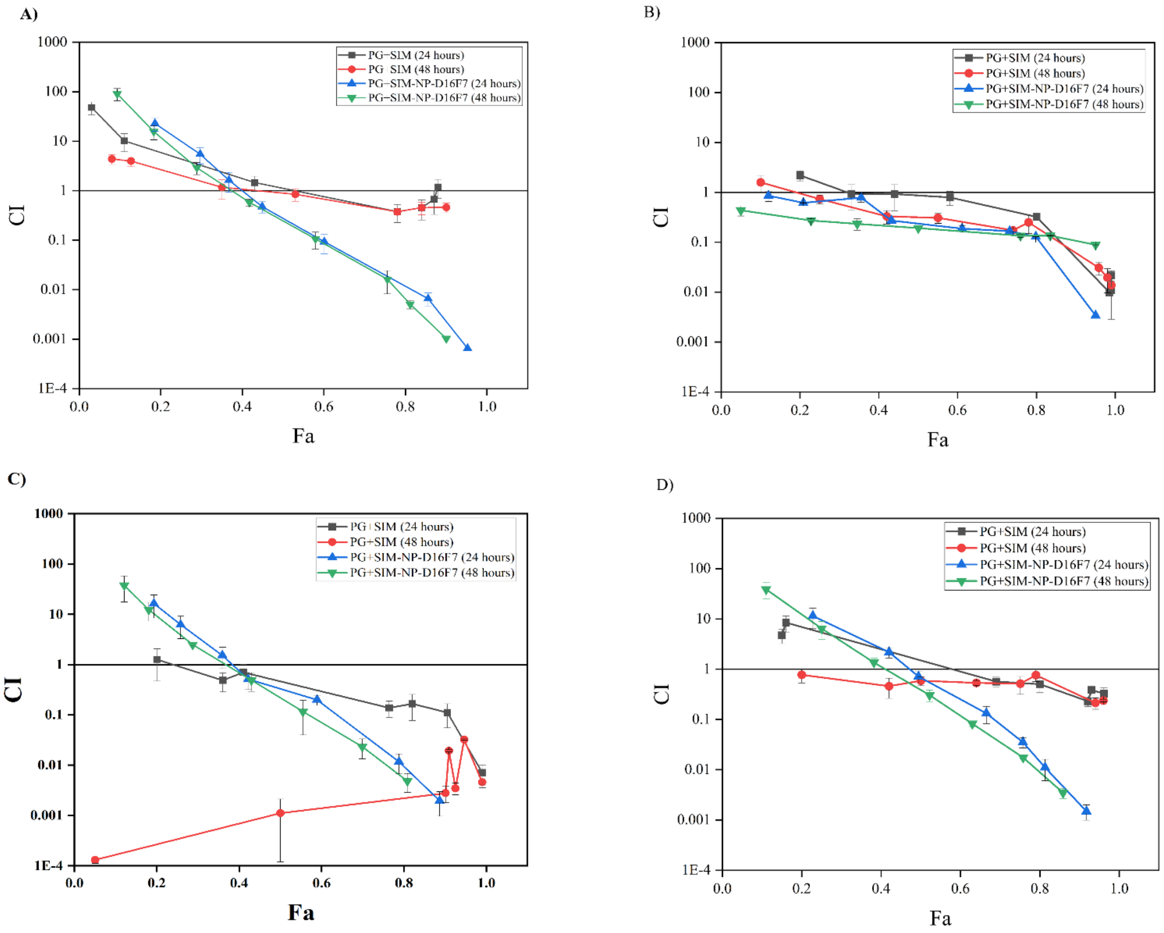

Figure. S5. Cellular uptake monitoring of PG+SIM, the PG+SIM-NP and PG+SIM-NP-D16F7 after addition to cell lines on A) MCF-7, B) LnCap, C) U87MG, and HSF. Images were taken after 30 min, 90 min, and 24 h of incubation.

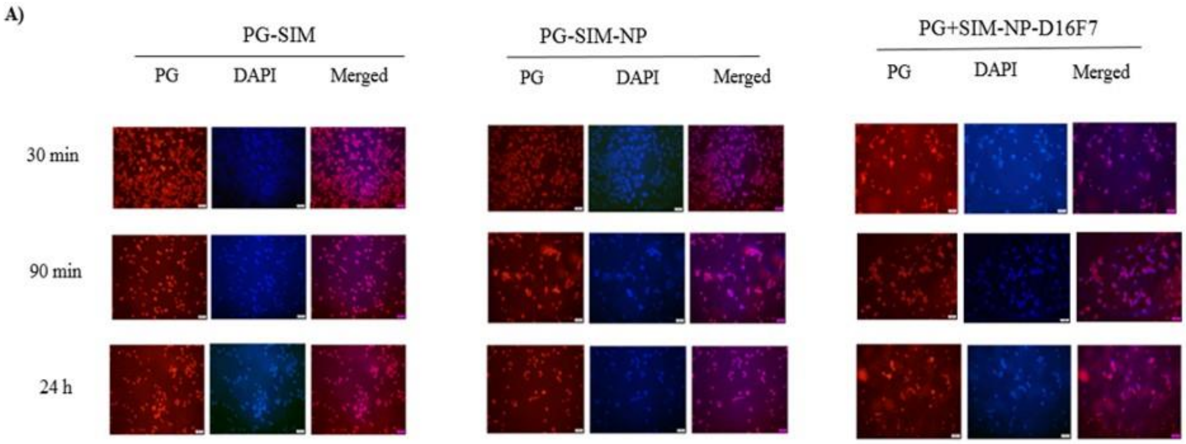

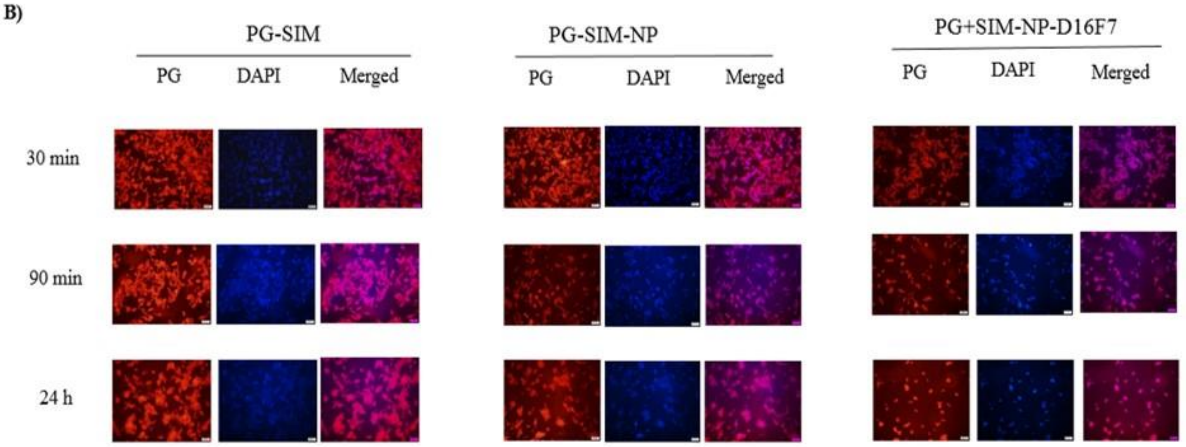

C)

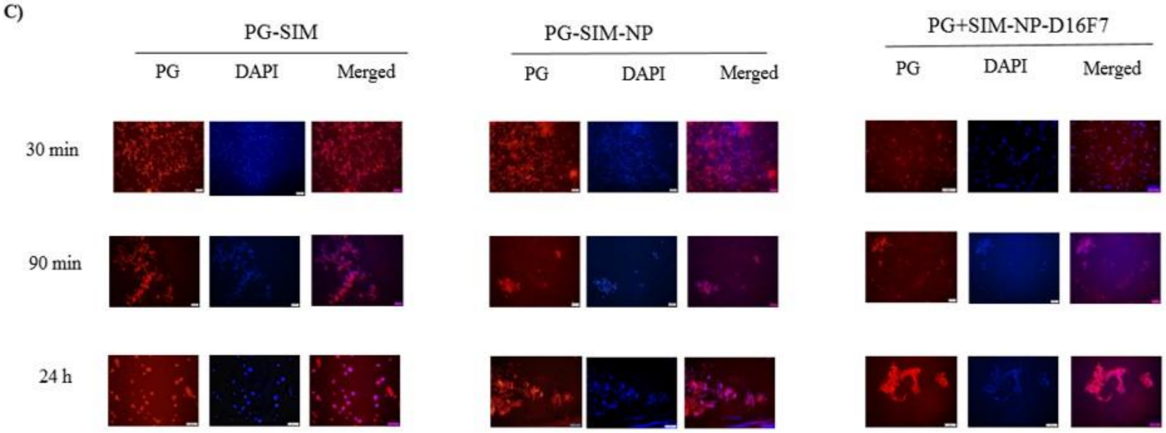
PG-SIM
PG
DAPI
Merged
PG-SIM-NP
PG
DAPI
Merged
PG+SIM-NP-D16F7
PG
DAPI
Merged
30 min
90 min
24 h

D)

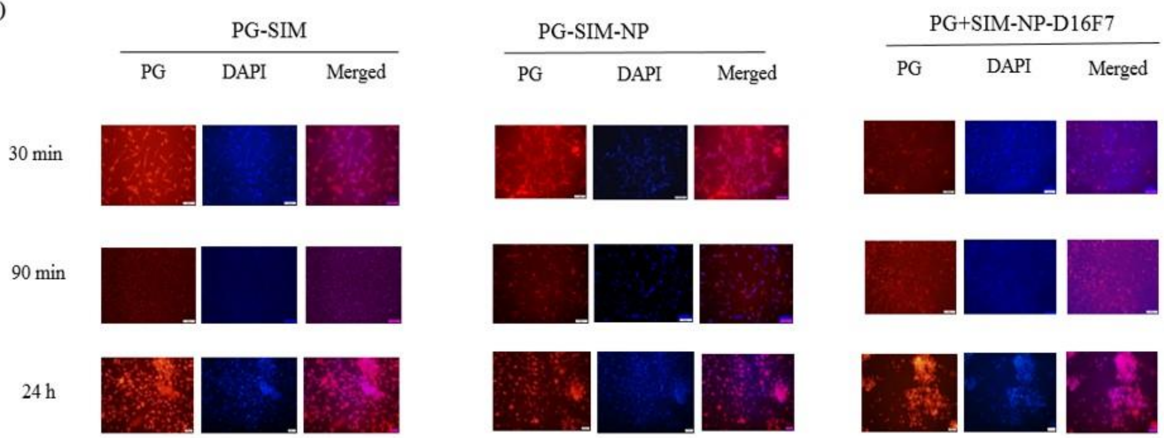
PG-SIM
PG
DAPI
Merged
PG-SIM-NP
PG
DAPI
Merged
PG+SIM-NP-D16F7
PG
DAPI
Merged
30 min
90 min
24 h
